# Integrin α_2_β_1_-targeting ferritin nanocarrier traverses the blood–brain barrier for effective glioma chemotherapy

**DOI:** 10.1186/s12951-021-00925-1

**Published:** 2021-06-13

**Authors:** Chiun-Wei Huang, Chia-Pao Chuang, Yan-Jun Chen, Hsu-Yuan Wang, Jia-Jia Lin, Chiung-Yin Huang, Kuo-Chen Wei, Feng-Ting Huang

**Affiliations:** 1 Center for Advanced Molecular Imaging and Translation (CAMIT), Department of Medical Research, Chang Gung Memorial Hospital, Linkou, Taiwan; 2 Department of Biochemical Science and Technology, College of Life Science, National Taiwan University, AC2-414, No.1, Sec. 4, Roosevelt Rd., Taipei, 106319 Taiwan; 3 Department of Neurosurgery, Chang Gung Memorial Hospital, Linkou, Taiwan; 4 Department of Neurosurgery, New Taipei Municipal TuCheng Hospital, New Taipei City, Taiwan; 5 School of Medicine, Chang Gung University, Taoyuan, Taiwan

**Keywords:** Ferritin, Integrin α_2_β_1_, Transferrin receptor 1, Receptor-mediated transcytosis (RMT), Blood–brain barrier

## Abstract

**Background:**

Ferritin, the natural iron storage protein complex, self-assembles into a uniform cage-like structure. Human H-ferritin (HFn) has been shown to transverse the blood–brain barrier (BBB) by binding to transferrin receptor 1 (TfR1), which is abundant in endothelial cells and overexpressed in tumors, and enters cells via endocytosis. Ferritin is easily genetically modified with various functional molecules, justifying that it possesses great potential for development into a nanocarrier drug delivery system.

**Results:**

In this study, a unique integrin α2β1-targeting H-ferritin (2D-HFn)-based drug delivery system was developed that highlights the feasibility of receptor-mediated transcytosis (RMT) for glioma tumor treatment. The integrin targeting α2β1 specificity was validated by biolayer interferometry in real time monitoring and followed by cell binding, chemo-drug encapsulation stability studies. Compared with naïve HFn, 2D-HFn dramatically elevated not only doxorubicin (DOX) drug loading capacity (up to 458 drug molecules/protein cage) but also tumor targeting capability after crossing BBB in an in vitro transcytosis assay (twofold) and an in vivo orthotopic glioma model. Most importantly, DOX-loaded 2D-HFn significantly suppressed subcutaneous and orthotopic U-87MG tumor progression; in particular, orthotopic glioma mice survived for more than 80 days.

**Conclusions:**

We believe that this versatile nanoparticle has established a proof-of-concept platform to enable more accurate brain tumor targeting and precision treatment arrangements. Additionally, this unique RMT based ferritin drug delivery technique would accelerate the clinical development of an innovative drug delivery strategy for central nervous system diseases with limited side effects in translational medicine.

**Graphic Abstract:**

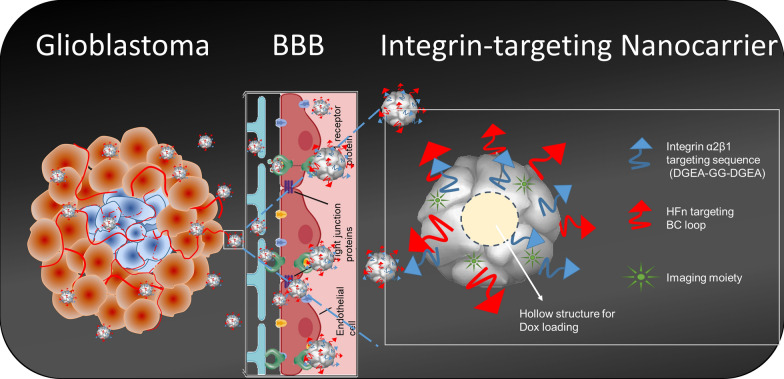

**Supplementary Information:**

The online version contains supplementary material available at 10.1186/s12951-021-00925-1.

## Introduction

Cancer is the second leading cause of mortality worldwide according to the WHO [1]. Tremendous efforts in biomedical research have been devoted to specific clinical therapies and new formulations of anticancer drugs. In conventional cancer treatment, chemotherapy drugs are usually delivered systemically via the circulatory system but are often prematurely released before reaching the tumors [2, 3]. The concentration of administered drug rapidly increases inside the body and elicits side effects or cytotoxicity, causing damage to normal tissues or cells [4]. Hence, for forthcoming precision cancer medicine development, the major goals are not limited to early diagnosis and phenotyping of the disease but also include improvements in drug delivery efficacy and the therapeutic index of drugs [5]. To enhance the efficacy of antitumor drugs and reduce side effects, developing an efficient delivery vehicle is the key.

Among the broad range of tumors, malignant tumors in the central nervous system (CNS) represent the greatest challenge for effective drug delivery due to the innate blood–brain barrier (BBB). Nanotechnologies may have great clinical potential in overcoming this formidable obstacle in traditional brain cancer treatment. Nanoparticles are purposely constructed on the nanometer scale, and due to their size, nanoparticles can penetrate more deeply into inflammatory sites, the epithelium and tumors. Moreover, nanoparticles can be loaded with various chemotherapeutic drugs and modified with targeting molecules to provide the targeted delivery of drugs to tumors [5]. Currently, liposomes and lipid-based polymers are the most common nanocarrier systems for targeted drug delivery. The diameters of such liposome particles vary from 25 nm to 2.5 μm [6]. As a drug delivery system, liposomes have several advantages, including biocompatibility, biodegradability, low toxicity and the ability to encapsulate both water- and lipid-soluble drugs [6]. However, liposomes as drug delivery platforms have not progressed as expected, and the major challenge or bottleneck is meeting pharmaceutical manufacturing regulations, current good manufacturing practices (cGMPs), on a large scale. Furthermore, the growing complexity of the addition of surface modifications with a variety of coating and/or targeting moieties and potential long-term toxicity present great obstacles in fulfilling quality assurance for clinical use.

On the other hand, ferritin, a ubiquitous and major iron storage protein found in most living organisms, is composed of 24 subunits that form a protein nanocage structure through 4-3-2 symmetry with external and interior cavity diameters of 12 and 8 nm, respectively [7–10]. Ferritin has several advantages for their development as promising drug delivery nanoparticles. First, the cage structure of ferritin self-assembles and is highly resistant to extreme environmental conditions. The ferritin cage disassembles when the pH becomes extremely acidic (pH = 1–2) or basic (pH = 11–13). When the pH value returns to neutral, the separated ferritin subunits are again able to self-assemble into the cage structure [11, 12]. Ferritin is also tolerant to high temperatures of up to 80 °C [13]. Ferritin from humans and some other eukaryotes have two kinds of subunit types: heavy (H)- and light (L)-chain ferritin, with molecular weights of 21 and 19 kDa, respectively [14]. The H subunit of ferritin (HFn) has a ferroxidase center within the bundle, which is composed of two nearby metal-binding sites and is responsible for the oxidation of Fe^2+^ into Fe^3+^. The L subunit of ferritin (LFn), without ferroxidase activity, facilitates mineralization of the iron in the cavity and electron transfer across the protein shell, supporting the ferroxidase activity of the H chain [15]. Importantly, extracellular ferritin interacts with cells by binding to transferrin receptor 1 (TfR1). TfR1 is overexpressed in several types of tumors and is a potential biomarker of cancer diagnosis [16]. Thus, ferritin has been developed as a diagnostic probe for molecular imaging detection in cancer [15, 17]. Most recently, the Fan et al. group demonstrated that ferritin composed purely of the H chain (HFn) has a high specific binding affinity for TfR1 [18]; thus, ferritin itself can overcome the BBB to reach glioma tumors through a receptor-mediated transcytosis (RMT) mechanism in a threshold dependent matter [18].

Integrins are a family of cell adhesion receptors that mediate intercellular or extracellular matrix interactions that are also upregulated and correlate well with the pathological grades in several tumor types [19]. Thus, integrin αvβ3 (targeted with RGD consensus peptide sequences) is currently the major target and has been applied extensively for molecular imaging and cancer therapy in clinical settings [20–24]. However, integrin α2β1, a less investigated receptor, binds to the collagen receptor and plays an important role in cancer progression and metastasis. For example, integrin α2β1 is overexpressed in various carcinoma tumors, including glioma, prostate cancer, breast cancer, lung cancer and colorectal cancer [19, 25–27]. Integrin α2β1 stimulates endothelial cells to express vascular endothelial growth factor (VEGF) [28]. The interaction between integrin α2β1 and VEGF can activate the MAPK/ERK pathway, leading to endothelial cell proliferation and angiogenesis in prostate carcinoma [29]. Furthermore, the binding of integrin α2β1 to type I collagen leads to the upregulation of the Rho-GTPase signaling pathway, which promotes prostate carcinoma to metastasize into bone [30].

Therefore, in this study, a genetic modified HFn with an integrin α2β1 targeting ligand (DGEAGGDGEA) [31–34] on the surface of nanocarrier was designed and characterized to traverse the BBB for glioma targeting and therapy. The proposed integrin α2β1-targeting HFn nanocarrier demonstrated specific binding with integrin α2β1 on U-87MG cells in in vitro experiments, with reduced binding to the 22Rv1 cells that served as a control, which have lower expression levels of both TfR1 and integrin α2β1. Furthermore, due to the biocompatibility, nanometer size, uniform structure and pH-dependent self-assembly of HFn, its hollow cavity loaded with doxorubicin (DOX) was utilized for chemotherapeutic delivery evaluation studies in vivo. These modified ferritin nanoparticles demonstrated a promising therapeutic index in both subcutaneous and orthotopic glioma tumor models, justifying their role as ideal candidates for nanoparticle drug delivery development for brain tumors and a variety of CNS diseases.

## Results

### Characterization of HFn and 2D-HFn

To enhance the tumor-targeting capability of HFn, the reported integrin α2β1-targeting peptide sequence DGEAGGDGEA [31–34] was designed to add to the N-terminus of HFn, and the modified HFn is referred to as 2D-HFn. HFn and 2D-HFn were purified by the same procedures, and physicochemical characterization of the two proteins revealed that 2D-HFn, as expected, has a slightly larger molecular weight than HFn (Additional file 1: Figure S2a). Both proteins had a uniform hollow spherical morphology (Additional file 1: Figure S2b) with dynamic average diameters of 12.1 nm (HFn) and 13.7 nm (2D-HFn), as measured and calculated by DLS (Additional file 1: Figure S2c). Collectively, these results showed that modified 2D-HFn shared similar structural features with HFn.

### Integrin binding specificity validation with biolayer (BLI) interferometry

**Fig. 1:**
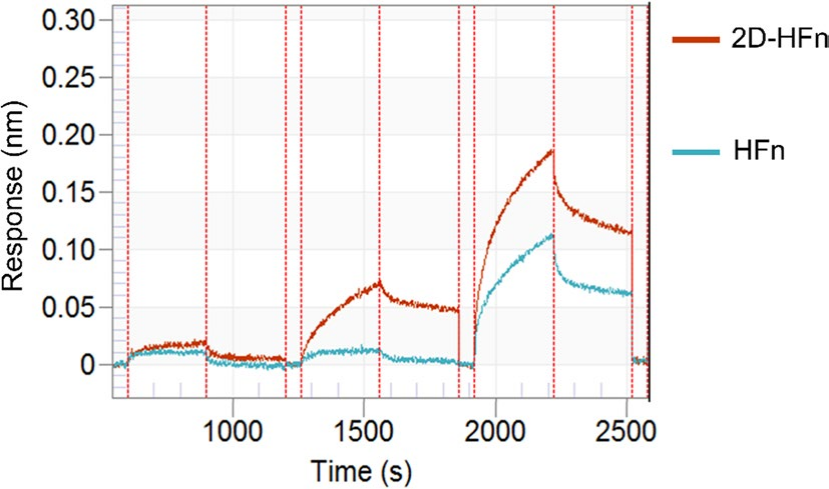
The validation of 2D-HFn and HFn binding to integrin α2β1 assayed with BLI. The integrin α2β1proteins were loaded to the sensor tip from a solution of 10 nM and then incubated with various concentrations of 2D-HFn and HFn in binding buffer with 2 mM Mg^2+^. The association and dissociation binding curves were monitored by eight-channel BLI system Octet RED96 and K_d_ values were calculated by Octet DataAnalysis software (9.0.0.6.)

The biolayer interferometry (BLI) is a fast, real-time, high throughput and label-free way of an optical analytical technique that analyzes the interference pattern of light reflected from wavelength shift (nm) caused by association and dissociation of a soluble macromolecule to an immobilized species; Interactions are measured in real time, providing the ability to monitor binding specificity, rates of association and dissociation, with high precision and accuracy. To elucidate and quantify the specificity and kinetic binding capability of integrin α2β1 with proposed 2D-HFn nanocarriers, BLI assays were performed (Fig. 1). In these experiments, the ratio of k_dis_/k_ass_ (rates of dissociation/association) was calculated to determine Kd to elucidate the binding affinity strength. The K_d_ value was determined with BLI for 2D-HFn and HFn as 9.32 ± 0.2 × 10^–7^ M (R^2^ = 0.95) and 3.63 ± 0.52 × 10^–5^ M (R^2^ = 0.87), respectively. Collectedly, the engineered 2D-HFn did demonstrate the higher binding affinity (200-fold) to integrin α2β1 than the naïve HFn protein. With these promising binding assay results, the in vitro cell binding and uptake experiments were proceeded to further validate whether 2D-HFn is suitable for integrin targeting drug delivery vehicle.

### In vitro receptor-mediated cell binding assays with HFn/2D-HFn

**Fig. 2:**
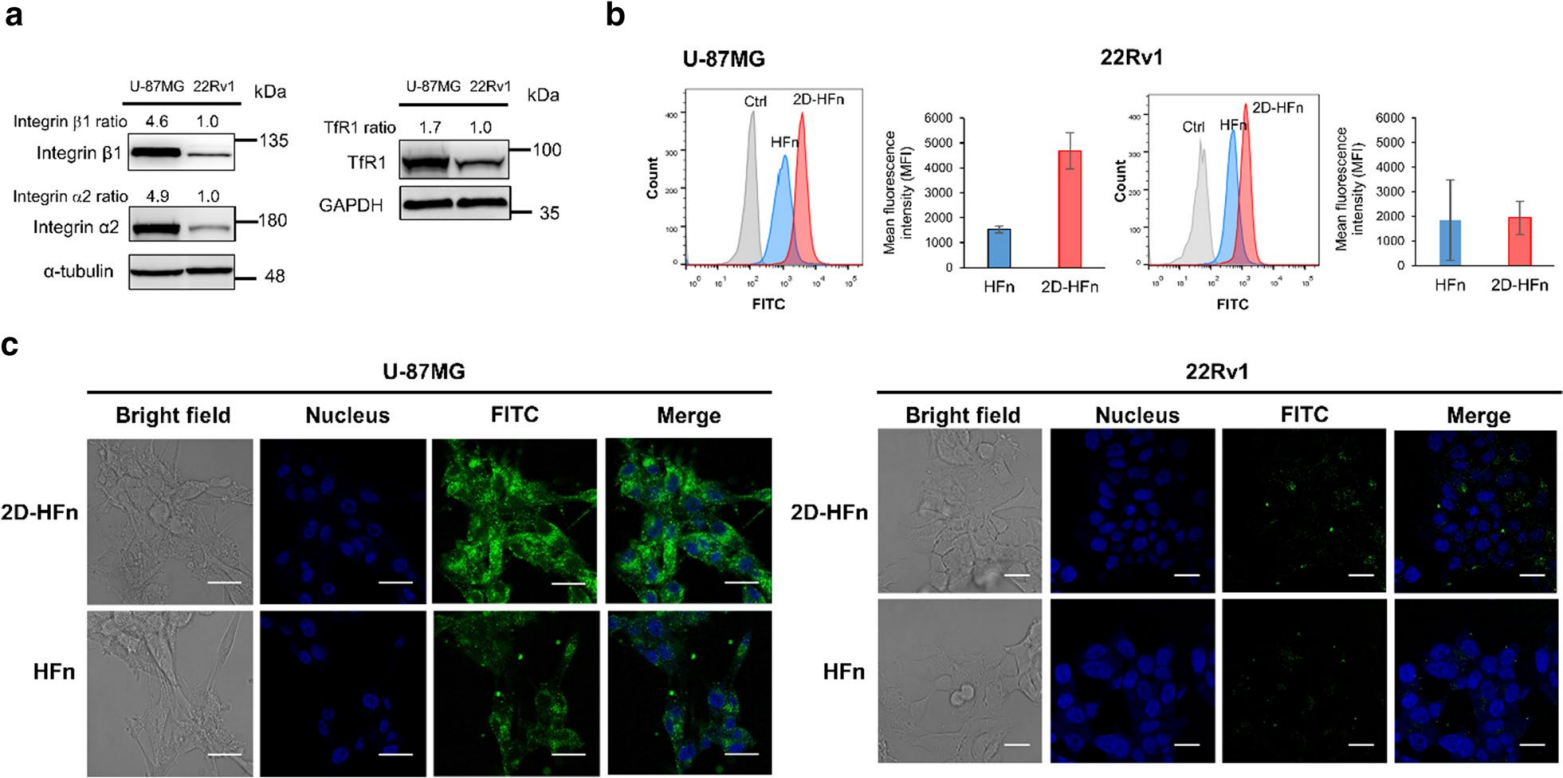
2D-HFn has a strong ability to bind to cancer cells. **a** High expression of integrin α2β1 and TfR1 in U-87MG cells, but not in 22Rv1 cells by western blotting using antibodies against integrin α2, integrin β1 and TfR1. **b** FITC-labeled 2D-HFn had a stronger cell binding ability to U-87MG cells than FITC-labeled HFn. The mean fluorescence intensity (MFI) of FITC-labeled 2D-HFn on U-87MG cells was approximately 3.6-fold greater than that of FITC-labeled HFn (N = 3; mean ± SEM). **c** After incubation of FITC-labeled 2D-HFn with U-87MG cells, 2D-HFn bound to the cell surface and entered into the cells. In contrast, few 2D-HFn molecules bound to the cell surface of 22Rv1 cells. Scale bar, 10 μm

To investigate cell binding specificity, one human prostate cancer cell line 22Rv1, and one human glioblastoma cell line, U-87MG, were selected. The malignant U-87MG cells had high levels of expression of integrin α2β1 and transferrin receptor 1 (TfR1), as demonstrated by western blot results (Fig. 2a). In contrast, 22Rv1 cells, with a relatively lower expression level of integrin α2β1 and TfR1 than the U-87MG cell line served as a control. FITC-labeled 2D-HFn and HFn were incubated with these cells for 30 min followed by flow cytometry analysis. FITC-2D-HFn demonstrated 3.6-fold higher fluorescent signals than FITC-HFn in U-87MG cells (Fig. 2b), indicating enhanced binding affinity due to integrin α2β1 targeting capability. However, the uptakes of FITC-2D-HFn and FITC-HFn in 22Rv1 cells were low, indicating the low binding specificity of the labeled proteins. Furthermore, the protein uptake distribution inside the cells was validated by confocal microscopy studies (Fig. 2c). After 30 min of incubation, FITC-2D-HFn quickly accumulated inside U-87MG cells but not 22Rv1 cells. Overall, the proposed integrin-targeting HFn nanoparticle showed specific tumor-targeting capability to the upregulated TfR1 and integrin α2β1 receptor in malignant lesions.

### In vitro stability of encapsulated DOX in 2D-HFn

**Fig. 3:**
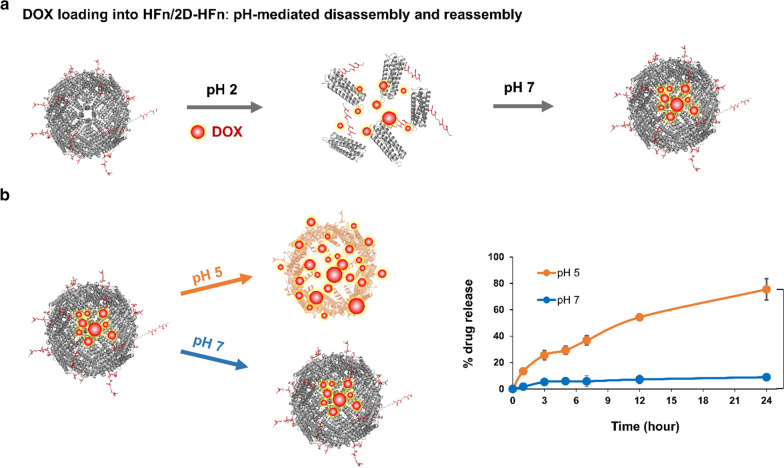
DOX was gradually released from DOX-loaded 2D-HFn in an acidic environment. **a** Encapsulation of DOX into 2D-HFn and HFn was performed by the pH-mediated disassembly and reassembly method. **b** A diagram showed the encapsulation of DOX into the ferritin complex and DOX was released at pH5 or pH7. DOX loaded inside DOX-2D-HFn at pH 7 was stable over 24 h, but 75% of the DOX was released from DOX-loaded 2D-HFn at pH 5 (**P < 0.01; paired *t*-test; N = 3; mean ± SEM)

We next determined whether 2D-HFn could serve as a nanocarrier for effective drug delivery into the selected cell lines in vitro. Therefore, the commonly used chemotherapeutic DOX was chosen for loading into FITC-2D-HFn (Fig. 3a). To analyze the encapsulation stability of DOX inside the 2D-HFn nanoparticle either under physiological conditions (pH 7), or in an acidic environment (pH 5), the DOX-2D-HFn nanoparticles were added to a dialysis device submerged in a pH 7 or pH 5 solution. The DOX concentration in the dialysis buffer was measured at various incubation time points (Fig. 3b). The encapsulated DOX inside 2D-HFn was quite stable at pH 7 for 24 h, and only 9% of DOX leaked into the surrounding solution. In contrast, at pH 5, DOX was gradually released into the solution over time, and 75% of DOX was released after 24 h of incubation. Therefore, under physiological conditions, most DOX remained stable inside the 2D-HFn nanoparticles but was gradually released under acidic conditions, such as those found in the tumor microenvironment or being endocytosed into the lysosome.

### The 2D-HFn nanoparticles carried and delivered DOX into cancer cells

**Fig. 4:**
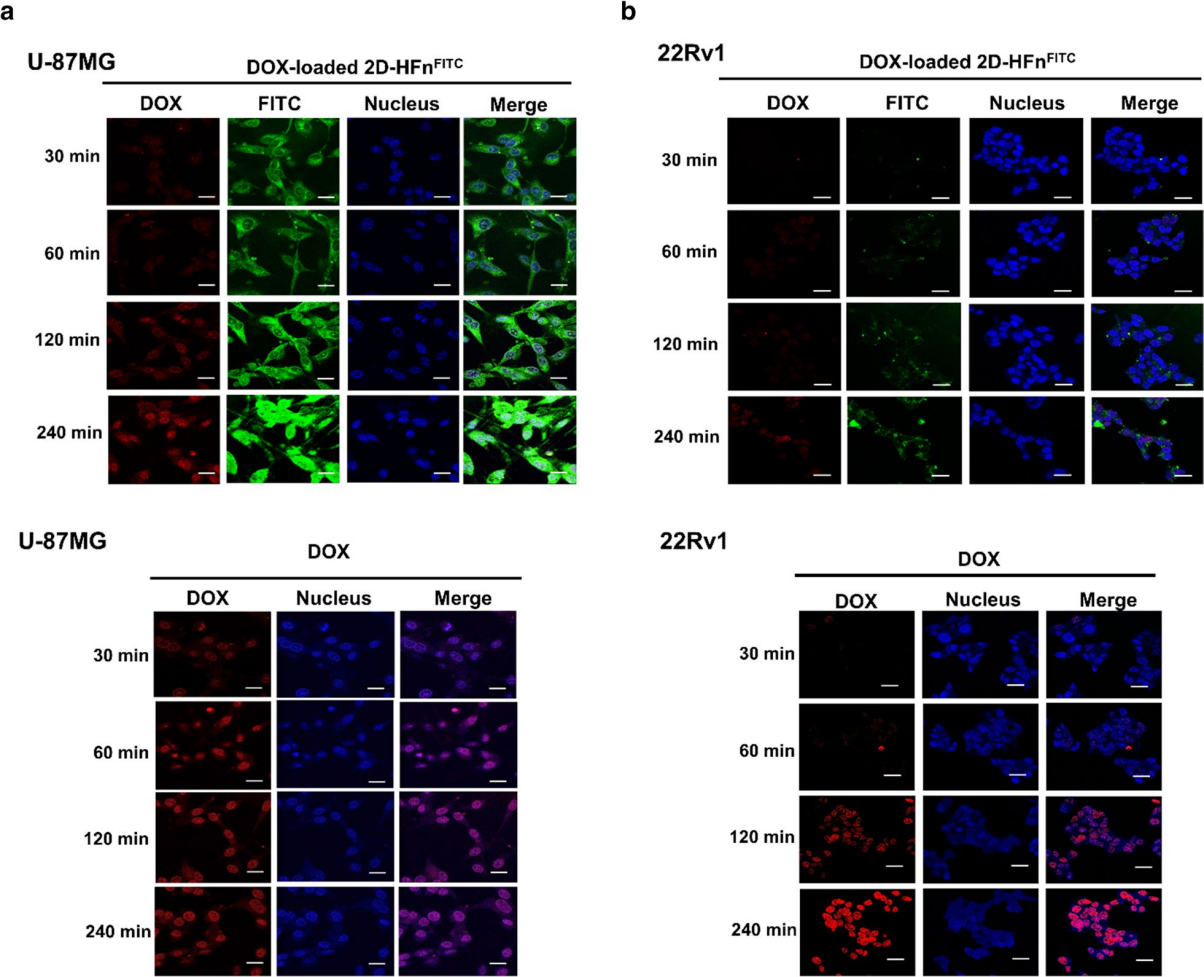
2D-HFn specifically delivered DOX to cancer cells that highly express integrin α2β1. **a** DOX-loaded 2D-HFn nanoparticles or DOX was incubated with U-87MG cells for various times. The free form of DOX was inside the cell nuclei after 30 min of incubation. However, DOX delivered via the DOX-loaded 2D-HFn nanoparticle was inside the cell nucleus after two hours of incubation and became obvious after four hours of incubation. The red signal represents DOX; FITC represents 2D-HFn. **b** The same procedure was conducted with 22Rv1 cells. Free DOX entered the cell nuclei after two hours of incubation. However, few DOX and 2D-HFn signals were observed, even after four hours of DOX-loaded 2D-HFn incubation. Scale bar, 10 μm

To determine the cellular uptake specificity, tumor cells were either incubated with either free DOX or FITC-2D-HFn-DOX for comparison. Intriguingly, free DOX was quickly taken up by both tumor cells and accumulated inside the nucleus within 120 min (Fig. 4). In contrast, specific FITC-2D-HFn-DOX uptake was observed only in U-87MG cells, which overexpress both integrin α2β1 and TfR1, indicating a receptor-mediated cell uptake mechanism. Based on confocal microscopy observations, FITC-2D-HFn-DOX first bound to the receptor and was gradually endocytosed by tumor cells, eventually slowly degrading in lysosomes; thus, controlled release of DOX was achieved, as evidenced by a gradual increase in the redshift fluorescent signal from DOX within the nucleus (Fig. 4a). However, the control 22Rv1 tumor cells only displayed negligible FITC and DOX signals throughout the experimental time period (Fig. 4b), further confirming the binding selectivity of FITC-2D-HFn-DOX as a drug carrier, which may reduce potential side effects.

### 2D-HFn nanoparticles efficiently delivered DOX into cancer cells and caused cell death with a lower dosage of DOX

**Fig. 5:**
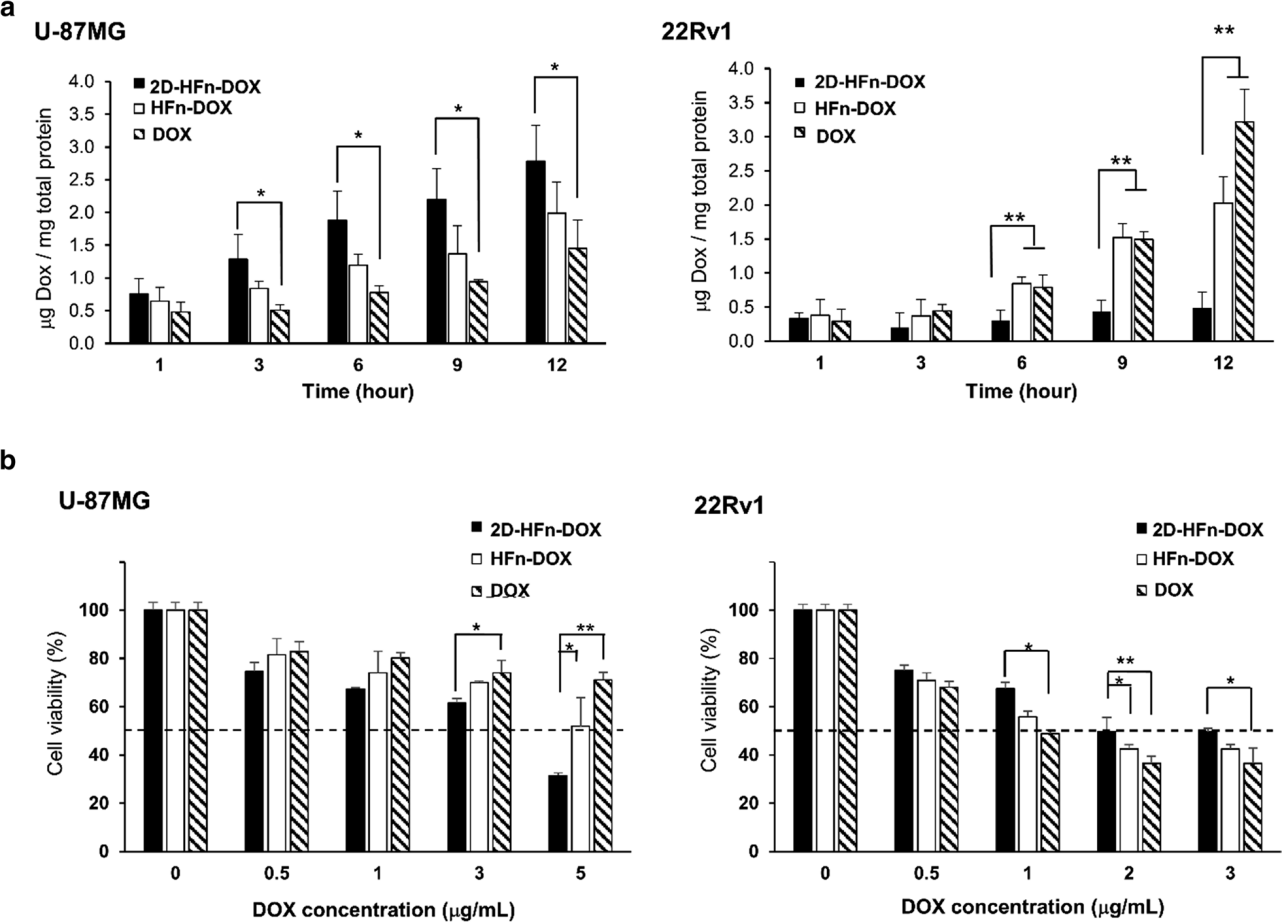
2D-HFn enhanced the cytotoxic effects of DOX on cancer cells. **a** Cellular DOX uptake via DOX-loaded 2D-HFn, DOX-loaded HFn or free DOX in U-87MG and 22Rv1 cells (*P < 0.05, **P < 0.01; one-way ANOVA; N = 3; mean ± SEM). **b** Cytotoxicity of DOX-loaded 2D-HFn, DOX-loaded HFn or free DOX in U-87MG and 22Rv1 cells. Dashed lines indicated 50% cell viability. (*P < 0.05, **P < 0.01; one-way ANOVA; N = 3; mean ± SEM)

To evaluate the treatment efficacy of DOX via carriers (2D-HFn or HFn nanoparticles) or in its free form, selected cancer cell lines were treated with the equivalent amount of DOX (2 μg/mL) via different carriers, and the delivered cellular DOX was longitudinally validated and quantitatively measured as mentioned (Fig. 5a). The U-87MG cells demonstrated the highest specific uptake of DOX through the 2D-HFn carrier, followed by HFn and free DOX. In particular, U-87MG cells demonstrated dominant DOX uptake through 2D-HFn within a short period of 3 h. On the other hand, 22Rv1 cells with negligible expression of integrin α2β1 showed the lowest DOX uptake after incubation with 2D-HFn-DOX throughout the entire experimental period. HFn-DOX demonstrated time-dependent cell uptake with the concentration of cellular DOX increasing over time but the uptake was still significantly less than that of free DOX after 12 h of incubation. Furthermore, the dose-dependent cytotoxicity of DOX was assessed via different carriers over 24 h in cell lines by MTT assay (Fig. 5b). In U-87MG cells, a lower dose of DOX in 2D-HFn was needed to reach the same cytotoxic effect as that of DOX in HFn or free DOX. Particularly, when cells were treated with 5 μg/mL DOX, 69% of cells were dead via 2D-HFn-DOX, but only 29% of cells were dead via free DOX, indicating that 2D-HFn-DOX has a higher therapeutic effect due to the RMT mechanism. However, fewer 22Rv1 cells were dead when the cells were treated with DOX-loaded 2D-HFn than when they were treated with DOX-loaded HFn or free DOX. Overall, the specific DOX uptake was confirmed by these in vitro cell uptake experiments, and the controlled drug delivery was consistent with the expression levels of the targeted receptors. As expected, the enhanced specific drug uptake boosted the therapeutic effects of DOX, conferring a potential treatment plan with a lower chemotherapy dosage with specific tumor uptake.

### Validation of the 2D-HFn tumor-targeting capability in vivo

**Fig. 6:**
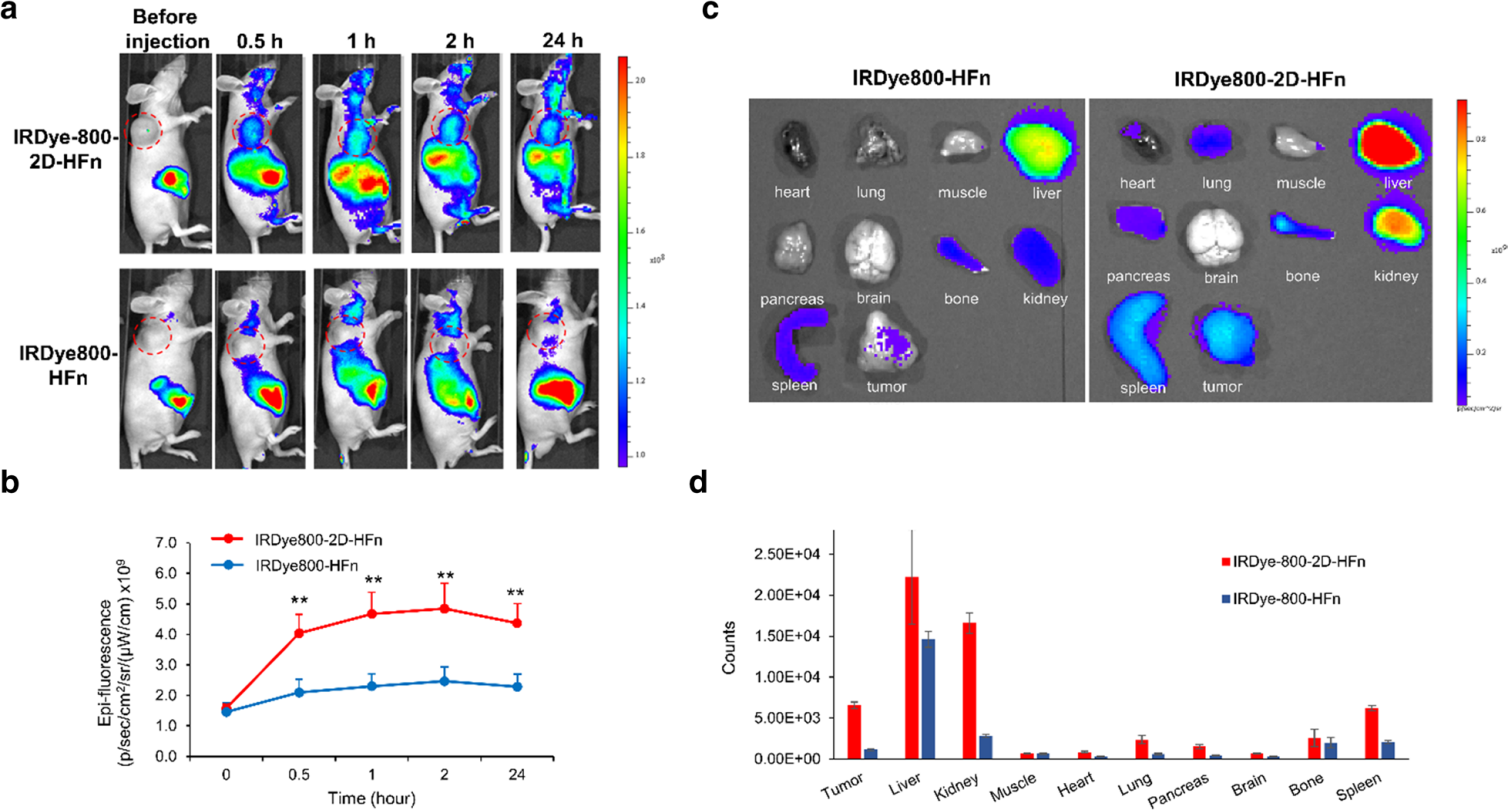
The tumor-targeting capability of 2D-HFn in U-87MG xenograft mouse models. **a** 2D-HFn had in vivo tumor-targeting capability in subcutaneous glioma mouse models. IRDye800-labeled 2D-HFn or IRDye800-labeled HFn was i.v. injected into U-87MG tumor-bearing mice, and the IRDye800 signal was acquired at various time points by an IVIS system. The dashed red circle represented the tumor location. **b** Quantitative values of the IRDye800 signal at different time points. Compared with HFn, the signal from 2D-HFn at the tumor site was approximately two-fold greater, and the tumor-targeting capability was enhanced (**P < 0.01; one-way ANOVA; N = 5; mean ± sd). **c** The biodistribution of 2D-HFn and HFn in U-87MG tumor-bearing mice. **d** The ROI signal analysis of the ex vivo organ imaging. The 2D-HFn signal in the tumors was stronger than the HFn signal in the tumors

To assess the tumor-targeting capability in vivo, HFn and 2D-HFn were labeled with IRDye800 for delivery tracking and biodistribution studies for real-time monitoring by optical IVIS imaging. IRDye800-labeled nanocarriers were injected into the tail vein of subcutaneous U-87MG tumor-bearing mice and longitudinally scanned at 0.5, 1, 2 and 24 h (Fig. 6a). Compared with IRDye800-HFn, IRDye800-2D-HFn demonstrated tumor-targeting imaging as early as 30 min after injection, and the signal continued to accumulate until 2 h and then reached a plateau. Additionally, during the whole experimental period (24 h), the fluorescent signal of IRDye800-2D-HFn remained approximately two-fold higher than that of IRDye800-HFn (Fig. 6b). After the last scanning time (24 h), the animals were euthanized, and the major organs (heart, lung, muscle, liver, pancreas, brain, bone, kidney and spleen) were excised for ex vivo fluorescent imaging studies (Fig. 6c). In addition to the high signals observed in the excretion-related organs, such as the liver, spleen and kidney, the tumor possessed the strongest signals, indicating the prominent tumor-targeting capability of the proposed 2D-HFn carriers. IRDye800-HFn showed a similar fluorescence signal pattern but to a much lesser extent (Fig. 6d), which may due to the quick washout rate of HFn. To validate the relationship among penetrated 2D-HFn nanoparticles, activated integrin α2β1 and tumor neovasculature, the ex vivo fluorescent imaging and histological tissue staining results were compared (Additional file 1: Figure S4).

### DOX-loaded 2D-HFn suppressed subcutaneous tumor growth with a low occurrence of toxic side effects

**Fig. 7:**
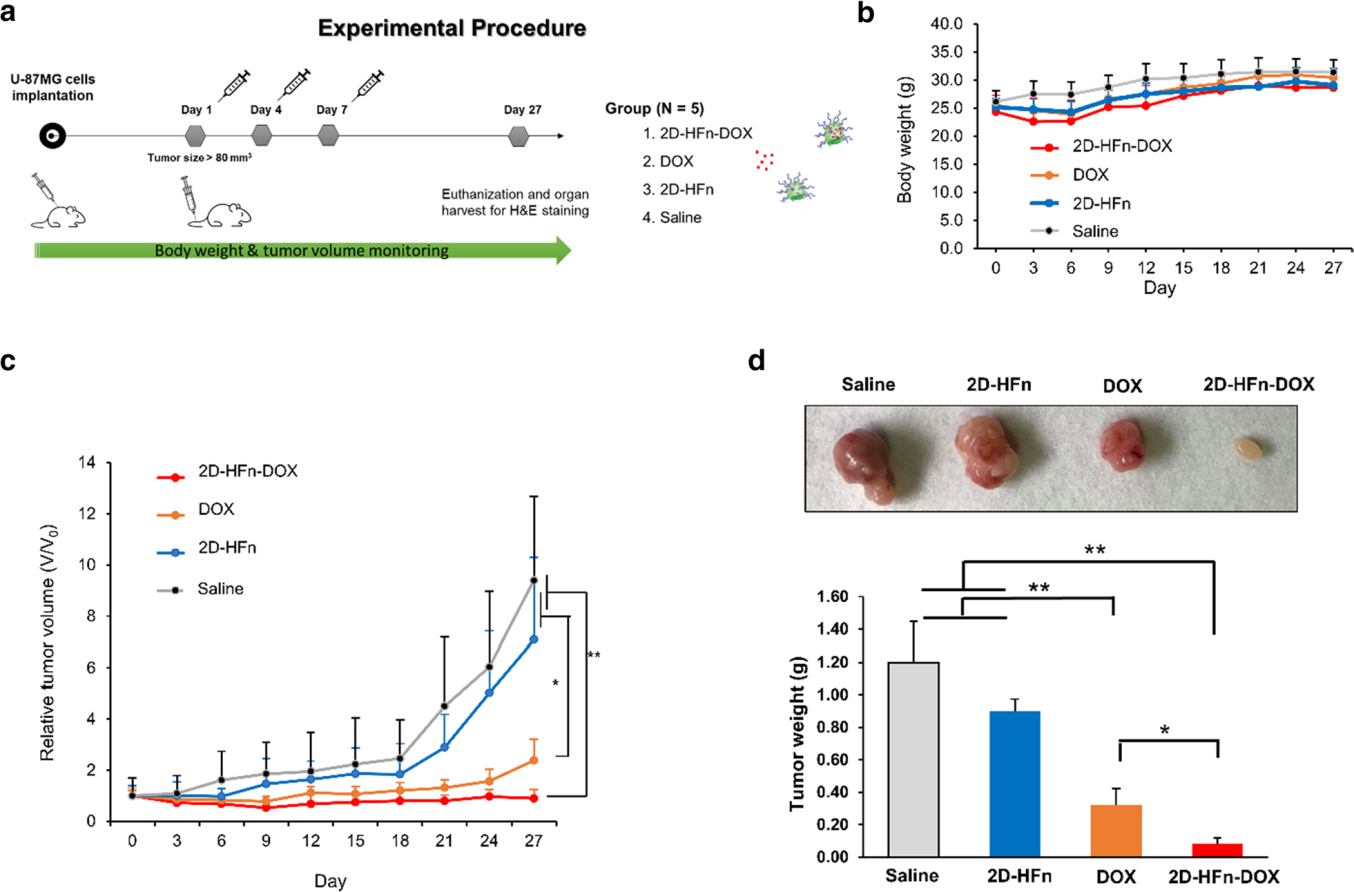
DOX-loaded 2D-HFn remarkably reduced tumor growth. **a** The experimental procedure of the therapeutic treatment on U-87MG tumor-bearing mice. Briefly, mice were implanted with U-87MG cells via subcutaneous injection. When the tumor size reached approximately 80 mm^3^, the mice were randomly divided into four groups and i.v. injected with various treatments every three days for a total of three injections. The four groups included the saline (control), 2D-HFn, DOX and DOX-loaded 2D-HFn (2D-HFn-DOX) groups. The body weights and the tumor sizes were measured every two days. **b** There was no significant difference in body weight among all groups during the procedure. **c** The tumor volumes of each group during the treatment. The average tumor volume of the DOX-loaded 2D-HFn group was remarkably smaller than that of the other groups (*P < 0.05, **P < 0.01; one-way ANOVA; N = 5; mean ± sd). **d** After treatment, the mice were sacrificed, and the tumors from all groups were dissected and weighed. Compared with the other groups, the average tumor weight of the mice treated with DOX-loaded 2D-HFn was the smallest (*P < 0.05, **P < 0.01; one-way ANOVA; N = 5; mean ± sd)

To assess the therapeutic effects of DOX-loaded 2D-HFn, subcutaneous U-87MG tumor-bearing animals were randomly divided into four groups and treated with saline, DOX, 2D-HFn, or DOX-loaded 2D-HFn three times via i.v. injection. The body weights and the tumor sizes of the mice were monitored once every three days after treatment (Fig. 7a). During the procedure, the body weights of the mice in each group were similar (Fig. 7b). The tumor sizes of the mice treated with DOX-loaded 2D-HFn were suppressed and remained the smallest among all groups (Fig. 7c), followed by the DOX-treated group. At the end of the experiment, the animals were sacrificed, and the tumors were dissected and weighed for direct comparison (Fig. 7d). The DOX-loaded 2D-HFn group demonstrated the most promising treatment results with the smallest tumor sizes and weights, followed by the DOX-treated group. On the other hand, both the saline and 2D-HFn groups demonstrated uncontrolled tumor growth, indicating no therapeutic effect. In addition, the levels of tumor growth inhibition in each group were consistent with the tumor section H&E staining results (Additional file 1: Figure S5). Furthermore, DOX-loaded 2D-HFn did not cause toxic side effects in major organs, such as the liver, heart and kidneys (Additional file 1: Figure S5). These results indicated that with encapsulation of the 2D-HFn nanoparticle, enhanced specificity and efficacy of chemotherapeutic drug delivery could be achieved without damaging normal tissues.

### 2D-HFn can traverse the BBB in in vitro and in vivo models

**Fig. 8:**
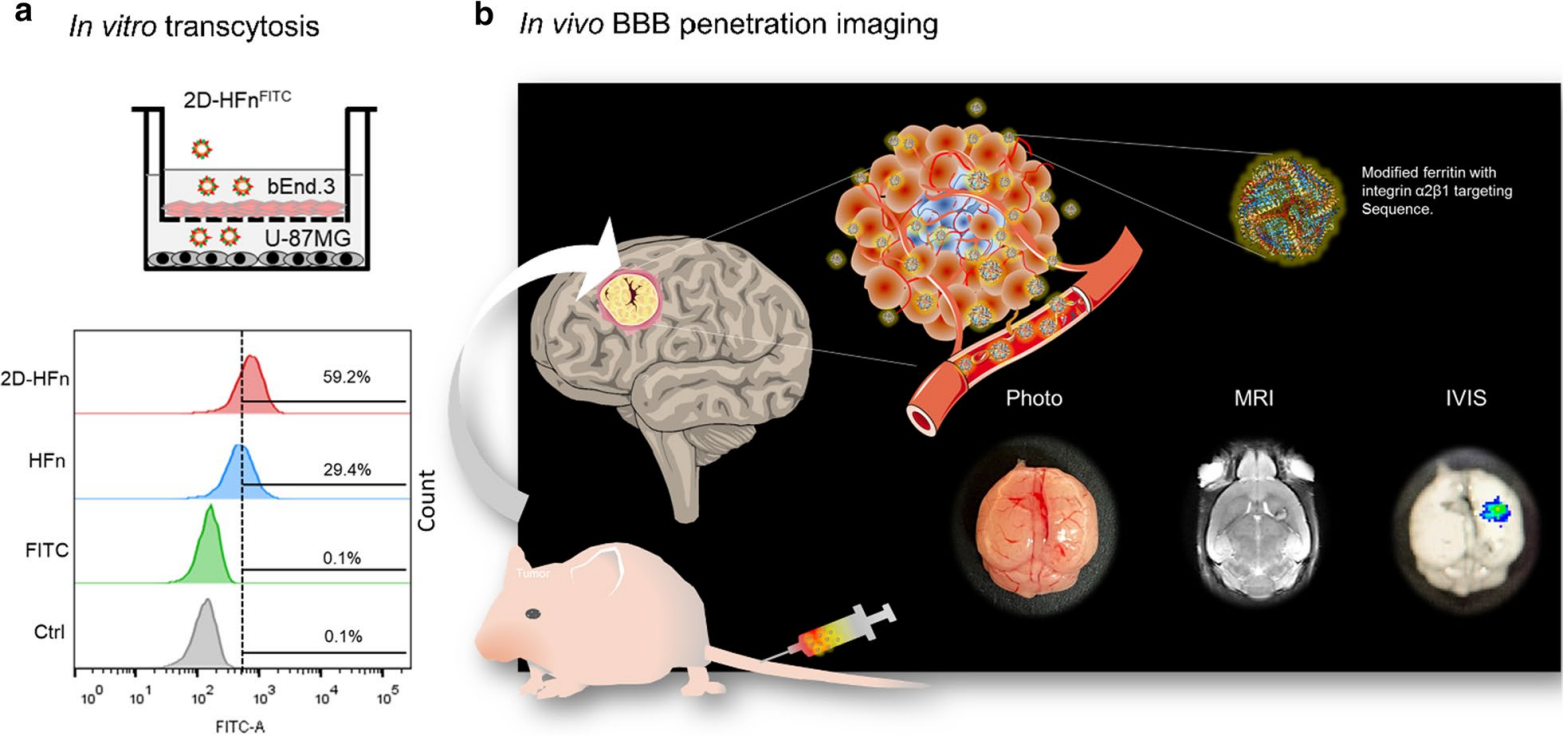
In vitro crossing of the BBB & in vivo tumor-targeting imaging. **a** The in vitro transcytosis experiment was performed to assess the abilities of HFn and 2D-HFn to cross the BBB. Briefly, bEnd.3 mouse brain endothelial cells were cultured in the upper chamber of a Boyden chamber to mimic the BBB. U-87MG cells were cultured in a 6-well plate. FITC, FITC-HFn, or FITC-2D-HFn was added into the medium of the upper chamber. After four hours of incubation, the U-87MG cells were harvested, and the FITC signal was analyzed by flow cytometry. No signal was detected in the FITC group. Approximately 29.4% of the cells were FITC-positive in the FITC-HFn group, indicating that FITC-HFn crossed the bEnd.3 cell layers and bound to U-87MG cells. In comparison, 59.2% of the cells were FITC-positive in the FITC-2D-HFn group, approximately two-fold greater than that of the FITC-HFn group. **b** 2D-HFn had a strong tumor-targeting capability in an orthotopic tumor model. IRDye800-2D-HFn was iv injected into the orthotopic U-87MG tumor model mice, and the tumor volume and location of IRDye800-2D-HFn were analyzed by MRI and IVIS imaging, respectively

As demonstrated by a published report [18, 35], HFn can actively cross the BBB through TfR1-mediated transcytosis; In addition, the contact binding sites between HFn and TfR1 were recently identified by Amédée des Georges and Beatrice Vallone with cryo-EM structure [36]. The HFn binding regions were among the external BC loop (R79, F81, Q83, K86, K87), the C-terminus of the C-helix (E116, K119. D123) and the N-terminus of the A-helix (Q14, D15, E17-A19, N21, R22, N25). Those involved amino acids were remained intact during the 2D-HFn construct development. Thus, to clarify whether the modified 2D-HFn still possesses a specific integrin binding capability as well as the ability to transverse the BBB, the in vitro transcytosis and in vivo cross BBB experiments were investigated. In the in vitro BBB transcytosis assay, both FITC-labeled HFn and FITC-labeled 2D-HFn effectively traversed bEnd.3 cells, but the control FITC dye alone failed to traverse the BBB (Fig. 8a). In comparison, the amount of integrin α2β1-targeted HFn transport across the BBB and bound to U-87MG cells was almost 2 times higher than that of HFn after four hours of incubation. To test whether 2D-HFn can cross the BBB to target glioma tumors in vivo, we administered IRDye-800-2D-HFn into orthotopic tumor-bearing animals (Fig. 8b). Although the tumor was extremely small (less than 6 mm^3^) compared with the subcutaneous xenografts, IRDye-800-2D-HFn still directly traversed the BBB and precisely bound to the tumor lesion with the high tumor-to-normal tissue ratio of 2. The fluorescence signal of the surrounding brain tissue was negligible, indicating robust 2D-HFn binding specificity. Together, these in vitro and in vivo results clearly demonstrated that 2D-HFn had an improved capability for controlled drug delivery and tumor targeting after traversing the BBB.

### Therapeutic index evaluation in an intracranial glioblastoma mouse model

**Fig. 9:**
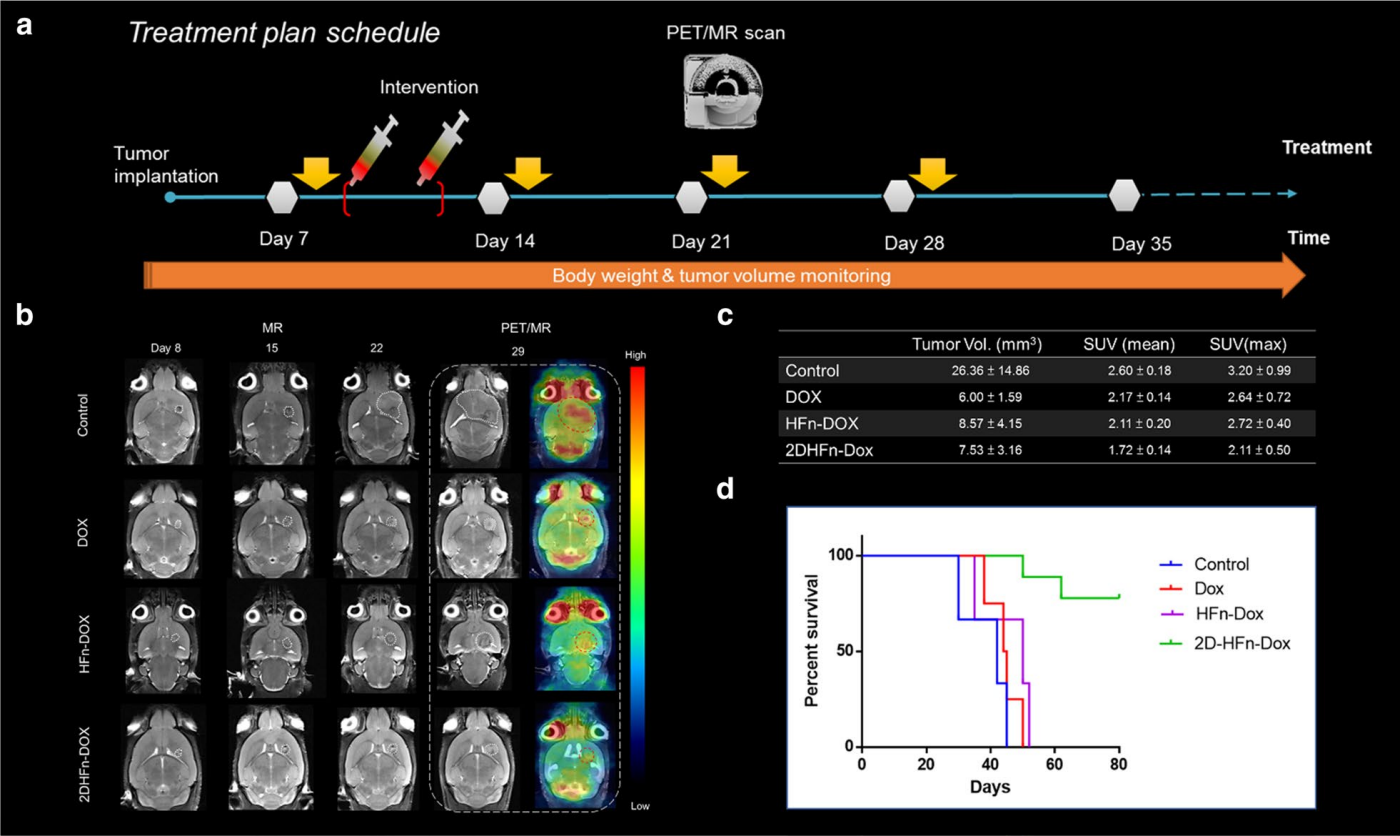
In vivo intracranial treatment, molecular imaging, and glioma mouse survival. **a** Treatment planning of the intracranial glioma tumor model. **b-c** In vivo PET/CT and MR imaging evaluation of glioma mice intravenously injected with saline (control), DOX, HFn-DOX or 2D-HFn-DOX and the quantitative analysis of anatomic tumor size and FDG uptake (N = 6). **d** Animal survival curves in the different treatment groups (Kaplan–Meier, *P* < 0.001)

With these promising proof-of-concept trans-BBB results, we moved forward to assess the therapeutic efficacy of drug-loaded 2D-HFn-DOX in the orthotopic glioma brain tumor model (Fig. 9). The implanted tumor growth was first validated by MR imaging (8 days after inoculation), and then the animals were randomly divided into four experimental arms: saline (Ctrl)-, free DOX-, HFn-DOX and 2D-HFn-DOX-treated groups. The treatments were conducted once every two days for a total of two treatments at the dose of 1 mg/kg DOX via intravenous injection. After 4 weeks, the tumor suppression effects were monitored by FDG-PET and MR imaging, in which PET imaging showed the viability of the surviving tumor cells and the MR image was used to provide anatomic tumor size information. As demonstrated in Figs. 9b, c, the tumor size and ^18^[F]-FDG uptake were highest in the Ctrl group, but the tumor sizes among the DOX, HFn-DOX and 2D-HFn-DOX groups were similar, demonstrating suppressed tumor growth upon treatment. However, in the FDG-PET imaging analysis, a significant reduction in ^18^[F]-FDG tracer uptake (approximately 17.28%) was observed in the 2D-HFn-DOX group compared with both DOX and HFn-DOX groups. This functional imaging information was further confirmed in the later survival analysis results (Fig. 9d), as 2D-HFn-DOX-treated animals outlived the mice in the DOX group by 40 days, until the end of the experiment period. This significant improvement in the median survival time was comforting and worth further detailed investigation of the sophisticated drug dosimetry, distribution and side effects for potential translational research designs.

## Discussion

Drug delivery systems have evolved from passive delivery to active targeting. Passive delivery primarily relies on the physical properties of particles, such as their size and net surface charge, and the leaky structure of the rapidly growing vasculature of tumor tissues for enhanced permeability and retention (EPR) effects [37]. In contrast, active targeting focuses on disease targeting and then promotes diagnostic signals and drug efficacy. Targeting capability focuses on the specificity and affinity of the targeting ligands specific to the biomarker of the disease. A successful drug delivery system should have certain requirements, including biocompatibility, nontoxicity, high disease-targeting specificity, and a high therapeutic index of the drug. Recently, various drug carrier development strategies and techniques have been developed to improve different aspects of drug delivery systems. The current and most promising delivery systems include liposomes, inorganic nanoparticles, and synthetic polymeric, dendrimer and protein cage architectures [38]. Nevertheless, these drug delivery systems have all encountered major obstacles in glioma therapy: difficulties in crossing the BBB and blood-tumor barrier (BTB) [39].

The BBB represents a formidable obstacle for its role as a protective interface between the CNS and peripheral circulating vasculature. Over the past decades, considerable effort has been put into developing active targeting nanocarrier that can cross the BBB and target glioma tumors. One promising strategy for transporting drugs across the BBB barrier is to target endogenous receptors, such as nicotinic acetylcholine receptors, TfR1, the insulin receptor, and low-density lipoprotein receptors for RMT [40]. Among these receptors, TfR1-mediated transcytosis is a popular choice for transporting drugs across the BBB and into the desired CNS regions [18, 35, 40]. The innate ligand of TfR1 (CD71) is human HFn through binding to the BC loop of HFn [36, 41], which is the primary reason why we chose to fabricate recombinant HFn for a glioma-targeting drug delivery system. HFn serving as a self-assembled modular nanocage is easily modified and can be conjugated on its surface with various molecules, including dyes, peptides, siRNA or antibodies [42]. In addition, the hollow HFn cavity can be loaded with various molecules, such as metal ions and drugs, for therapeutic purposes. As thoroughly elucidated by Fan et al. in 2018, the HFn can cross the BBB with TfR1-mediated RMT mechanism, and the HFn-encapsulated DOX significantly reduce the glioma tumor burden and improve the median survival times [18]. However, the current main drawback of ferritin-based carriers is their lack of drug encapsulation efficiency, and the average DOX loading ratio of HFn in the current study was approximately 11–16% with the use of the pH jump method to encapsulate drugs, the variation in drug loading efficiency between batches is very large (Additional file 1: Figure S3). Intriguingly, modified 2D-HFn demonstrated the ultra-high drug encapsulation efficiency of 61.7%, which is over 5 times higher than that of HFn and the drug loading molecules per protein cage can even reach 458. Ling Ahang et al. also reported that the higher affinity between paclitaxel (PTX) and HFn is the reason why the encapsulation efficiency of PTX-loaded HFn is higher than encapsulation with DOX, curcumin and Olaparib [35]. Thus, the higher loading ratio of DOX with 2D-HFn might be attributed to the modified integrin α2β1 targeting sequence, which possesses multiple carboxyl groups that may play a role in an ionic interaction between DOX and 2D-HFn. Furthermore, in in vitro cell transcytosis assay (Fig. 8a), the 2D-Hfn demonstrated over twofold BBB traverse and tumor retention efficacy, indicating that the proposed integrin α2β1 targeting 2D-HFn did have the leverage advantage over naïve HFn nanocarrier, in terms of the ideal drug delivery system. Therefore, in our study, the orthotopic glioma tumors were almost eradicated and showed no relapsed (over 80 days) and demonstrated the superior therapeutic benefits especially when compared with free DOX and HFn-DOX, in which the median survival day is 44.5 and 50 days, respectively. Overall, the detailed mechanism of how this high drug encapsulation of DOX within 2D-HFn can be achieved is under investigation. Moreover, this unique feature will be exploited to load a variety of chemotherapy drugs if the loading efficiency and targeting can be maintained, which may soon pave a new avenue for ferritin-based therapeutics in the fast-growing field of precision medicine and clinical applications.

The other limitation of ferritin-based drug delivery systems is that the extreme pH change may cause damage to the desired cargo and that the process is not fully reversible; the correctly folded protein can only reach 50–60% integrity from the disassociated protein subunits. Therefore, HFn can associate/dissociate under physiological conditions, such as *Archaeoglobus fulgidus* HFn (AfFt) [43], which should be an ideal encapsulation protein carrier. However, due to the lack of intrinsic TfR1 selectivity may make it not suitable for glioma targeting therapy, but adopted with other tumor-targeting capabilities, such as integrin targeting moieties might be utilized for tumors across a broad spectrum. The preparation of such versatile AfFt as a therapeutic agent will be complex and challenging, but worth pursuing in follow-up studies.

On the other hand, many types of solid tumors originate from epithelial cells, and epithelial integrins are generally retained or upregulated in the cancerous state, such as the integrins α6β4, α6β1, αvβ3, αvβ5, α2β1, and α3β1 [19]. Therefore, many studies have adopted strategies to target integrins or interfere with integrin expression for cancer diagnosis and therapy, respectively. Among all integrins, integrin αvβ3 is well known to be upregulated at the invasive front of malignant tumor types and during their angiogenic blood vessel growing process but weakly expressed on normal tissues and quiescent blood vessels. Due to its strong correlation with angiogenesis, the hallmark of tumor staging and phenotype, integrin αvβ3 is the most widely adopted biomarker for tumor-targeting agent development. However, in addition to the wide binding specificity of a variety of integrins, it has been reported that repeated administration of integrin αvβ3-targeting RGD peptides would most likely trigger immunogenicity [44]. Furthermore, the distribution and number of the targeting ligands, Arg-Gly-Asp (RGD), modified on the ferritin nanocarrier surface have the significant impact on efficacy of active tumor targeting [45]. The surface ligand pattern should be optimized to fit the receptor clustering feature to achieve the best tumor targeting. Thus, the integrin α2β1 targeting 2D-HFn was proposed and significant improvement in the consequent in vitro and in vivo tumor targeting studies, indicating that the precise functionalization of targeting ligand is crucial for better efficacy in ferritin based cancer nanomedicine and worth of further investigation.

**Fig. 10:**
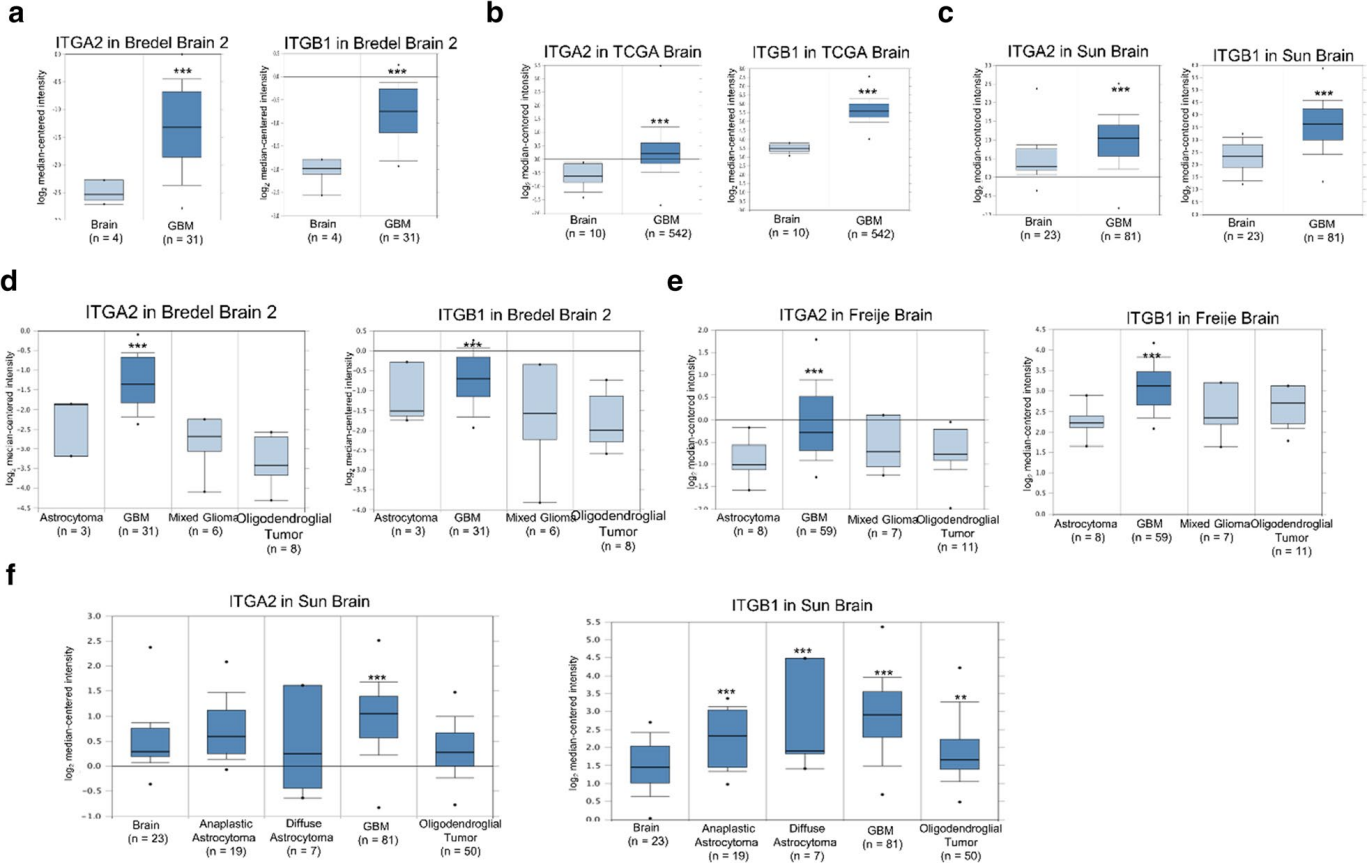
Integrin α2 (ITGA2) and integrin β1 (ITGB1) expression were up-regulated in glioblastoma in clinical cancer samples in three datasets from the Oncomine online microarray database. The mRNA level of integrin α2 (ITGA2) and integrin β1 (ITGB1) in clinical brain tumor tissues and normal brain tissues were acquired and analyzed from the Oncomine online microarray database. A box-and-whisker plot that represents ITGA2 and ITGB1 expression. The horizontal top and bottom lines of each box represented the 75th and the 25th percentile, respectively. The band in the box is the median value. Horizontal lines above and below the box represented the 90th and the 10th percentile, respectively. The dots above the 90th percentile and below the 10th percentile represented the maximum and minimum values, respectively. **a**–**c** From three brain datasets, ITGA2 and ITGB1 are up-regulated in GBM tissue specimens in comparison to normal tissues. **d**–**f** From three brain datasets, ITGA2 and ITGB1 are up-regulated in GBM tissue specimens in comparison to other subtypes of brain tumor tissue specimens and normal brain tissue specimens. Detailed information was listed in Additional file 1: Table S1. ** *P* < 0.01, *** *P* < 0.001

Furthermore, clinically upregulation of integrin α2β1 was also observed in advanced glioma tumors (Fig. 10) and the majority of malignant tumors, such as non-small-cell lung cancer (NSCLC), prostate cancer, breast cancer and pancreatic cancer (Additional file 1: Figures S6–8 and Table S2–3). The most common and deadly glioma tumors include astrocytomas (~ 50% of primary brain tumor), oligodendrogliomas squamous cell carcinoma (~ 4% of primary brain tumor), ependymomas (2–3%) and various other subtypes [46]. The glioblastoma (GBM), the grade IV astrocytoma, accounts for over 50% of the incidence of astrocytomas and has median survival of less than 2 years. Compared with normal brain tissue, ITGA2 and ITGB1 mRNA expression were up-regulated in clinical GBM specimens in several datasets, being increased by 1.432- to 4.437-fold of its expression in normal tissue specimens (Fig. 10a–c & Additional file 1: Table S1). Among all subtypes of brain tumors, the clinical data-mining analysis also indicated that ITGA2 and ITGB1 expression levels were significantly higher in GBM than other subtypes of brain tumors (Fig. 10d–f & Additional file 1: Table S1). Collectively, the developed HFn-based integrin α2β1-targeting nanoparticle system appears to be particularly promising for the most challenging treatment arrangement in glioma tumors but may also possess high clinical value for a wide range of tumor types.

Lastly, ferritin by itself has been widely applied in molecular imaging due to its multifunctional properties, such as easy genetic engineering of the fused targeting moieties and metal ion mineralization inside nanocages. The proposed 2D-HFn could be a potential theranostic agent due to its unique dual-targeting capability and promising in vivo therapeutic results. Radiolabeling the currently proposed 2D-HFn nanocarrier with diagnostic isotopes (Ga-68, Cu-64, Zr-89 and I-124) or therapeutic isotopes (Lu-177 and Y-90) can be easily achieved with facile chelator conjugation onto the ferritin surface with free amine or thiol groups. Thus, multifunctional ferritin could be a valuable tool and provide a robust strategy for theranostic agent development due to its multimeric nature, nanoscale particle size, self-assembly, drug loading capability and unique natural TfR1 targeting feature.

In all, the proposed integrin α2β1 targeting HFn drug carriers present several advantages for facilitating clinical translation as a ‘magic bullet’ drug delivery system in the near future. Firstly, the poly-carboxylic group of the –DGEA- targeting sequence not only increased the overall hydrophilicity of the HFn, but enhanced the interaction and loading efficiency with drugs, in that most drugs are encapsulated in HFn through charge interaction. Secondly, just like the innate TfR1 targeting of HFn is threshold dependent [18], the binding affinity among integrins and their ligands are also usually low and need the multiple active biding of clustering integrins, thus only when both receptors are up-regulated would cause significant endocytosis uptake of drug loaded HFn. The integrin-targeting capability of proposed 2D-HFn may provide the possibility of fine tuning the targeting efficacy and specificity of naïve HFn and minimize the off target side effects in normal tissues. The maturity of the proposed ferritin nanocage drug delivery system should have a great impact on the rapidly developing field of nanomedicine and precision medicine.

## Conclusion

Together, these in vitro and in vivo results clearly demonstrated that the proof-of-concept 2D-HFn drug carriers had an improved capability to traverse the BBB for controlled drug delivery and tumor targeting therapy. The particulate nature of the proposed ferritin particle was proved to create an ideal platform for targeted drug delivery coupling with dual tumor markers (integrin α_2_β_1_ and TfR1) for specific drug delivery and molecular imaging monitoring capability. It should ensure the development in this rapidly advancing area of theranostic research in drug delivery, both for a better understanding of fundamental pharmacokinetic mechanisms of proposed nanocarriers, and their applications to evaluate disease course and therapeutic efficacy at the earliest stages of treatment in in vivo models. However, the further investigation of the combined targeting and therapeutic efficacy of different drug molecules in the proposed HFn nanoparticle platform is still needed. This will include a process development on radiochemistry, molecular biology, bioengineering, nanotechnology and imaging diagnosis evaluation for potential translation and clinical applications.

## Materials and methods

### Plasmid construction

Ferritin cDNA was amplified from the cDNA library of U-87MG cells. According to the published sequences of the National Center for Biotechnology Information (NCBI) database, the primers were designed to contain the ferritin sequence along with the NcoI and XhoI sites for amplification and cloning into the pET28 plasmid. For the construction of the modified ferritin plasmid with integrin α2β1 targeting peptide (2D-HFn plasmid), the targeting peptide sequence DGEAGGDGEA [31–34] was designed adding at the N-terminus of ferritin, and the derived nucleotide sequences were designed for incorporation into the primer for amplification. The forward and the reverse primers for cloning the HFn plasmid were 5ʹ-CCATGGGAACGACCGCGTCCACCTCGCAGGTG-3ʹ and 5ʹ-CTCGAGTTAGCTTTCATTATCAC TGTCTCCCAG-3ʹ, respectively. The forward primer for cloning the 2D-HFn plasmid was 5ʹ-CCATGGGCGACGGAGAGGCAGGAGGCGACGGCGAAGCCGGAG GCGGAGGCACGACCGG-3ʹ. The HFn and 2D-HFn plasmids were confirmed by DNA sequencing.

### Protein expression and purification

The HFn and 2D-HFn plasmids were transformed into *E. coli* BL21 (DE3). For expression, the bacteria were cultured and induced with 0.2 mM isopropyl β-d-1-thiogalactopyranoside (IPTG) for 3 h. After cell disruption, the bacterial lysate was heated at 80 °C for 10 min. After centrifugation, the cell lysates were precipitated with ammonium sulfate (60% saturation). Subsequently, the cell lysate was further purified by size exclusion chromatography with a Sephacryl S-300 HR column (GE Healthcare, Aurora, OH, USA) (Additional file 1: Figure S1). After concentration and buffer exchange to phosphate buffer, pH 7.5 (PBS), the purified proteins were stored at -80 °C.

### Transmission electron microscopy (TEM) and dynamic light scattering (DLS) Analyses

For TEM analysis, the protein sample (0.5 mg mL^−1^) was transferred to carbon-coated 200-mesh copper grids. The grids were stained with 4% Uranyl Acetate. Excess stain was removed with filter paper, and the sample was air-dried for overnight. The images were recorded with a Hitachi HT7800 transmission electron microscope operating at 100 kV. For DLS analysis, the particle sizes of the protein samples (1 mg mL^−1^) were measured with a Malvern ZetaSizer Nano Z-S instrument at 25 °C.

### DOX loading into HFn/2D-HFn

The procedure for DOX encapsulation into HFn and 2D-HFn was performed by using the pH-assembly method. Briefly, 1 mg of protein was dissembled by adding 1 M HCl. Next, 20 μL of the DOX solution (10 mg/mL) was added followed by incubation at 22 °C for 15 min in the dark. Protein was then reassembled by adjusting the pH to 7.4 with the addition of 1 M NaOH and incubated at 22 °C for 15 min. Free DOX was removed by using a centrifugal filter with a 50 kDa cutoff (Merck, Burlington, MA, USA). The DOX concentration was determined by absorbance spectroscopy at 492 nm. The protein concentration was determined by DC™ protein assay (Bio-Rad, Hercules, CA, USA). The loading efficiency and yield were calculated according to the following formulas: loading efficiency = [weight of DOX loaded in protein/total weight of added DOX] × 100%; and yield = [weight of final protein /total weight of added protein] × 100%.

### Drug release test

The appropriate amount of 2D-HFn-DOX was loaded into a Slide-A-Lyzer dialysis device with a 10 kDa cutoff (Thermo, Waltham, MA, USA) and incubated in 15 mL of PBS (pH 7.4) or acetate buffer (pH 5) at 37 °C in the dark. At various time points, 0.3 mL of buffer was taken and replaced with fresh buffer. The amount of DOX in the 0.3 mL of removed buffer was measured and calculated by fluorescence spectrometry (λex = 490 nm, λem = 590 nm) based on the standard curve of DOX.

### Cell culture

The U-87MG, 22Rv1 and bEnd.3 cell lines were cultured in an incubator with 5% CO_2_ at 37 °C. U-87MG cells were cultured in MEM supplemented with L-glutamine, 10% fetal bovine serum (FBS), 100 units/mL penicillin–streptomycin, 1 mM sodium pyruvate, and 0.1 mM nonessential amino acid (NEAA). 22Rv1 and bEend.3 cells were cultured in DMEM supplemented with L-glutamine, 10% FBS, and 100 units mL^−1^ penicillin–streptomycin. All reagents were purchased from Gibco (Thermo Fisher, Waltham, MA, USA). U-87MG, 22Rv1 and bEend.3 cells were purchased from the ATCC (USA). Cell line authentication was performed using the GenePrint 24 system (Promega, Madison, WI, USA) and analyzed with an ABI PRISM 3730 Genetic Analyzer and GeneMapper Software V3.7. Cells were tested twice a year and found to be negative for mycoplasma using an EZ-PCR Mycoplasma Test Kit (Biological Industries, Israel).

### Western blotting

Cells were disrupted with RIPA buffer, and the total protein of the cell lysates was resolved by 12% SDS-PAGE. After electrophoresis, the proteins were transferred to a PVDF membrane, and the membrane was incubated with antibodies. The signal of the membrane was developed with PierceTM ECL Western blotting Substrate (Thermo, Waltham, MA, USA) and analyzed with a UVP Biospectrum™ imaging system (UVP, Upland, CA, USA). The primary antibodies against integrin α2, integrin β1, GAPDH and α-tubulin were purchased from GeneTex (Irvine, CA, USA). The primary antibody against ferritin and TfR1 were purchased from Santa Cruz (Dallas, TX, USA) and Cell Signaling (Beverly, MA, USA), respectively.

### Biolayer interferometry

The eight-channel BLI system Octet RED96 (ForteBio, Menlo Park, California) was used to conduct the titration binding experiments at room temperature with shaking at 1000 rpm. Due to the low binding affinity of integrin α2β1, the Anti-Penta-HIS tips (“SSA,” ForteBio, Pall Life Sciences) were used for His-tagged integrin α2β1 protein (ACROBiosystems, Newark, DE, USA) loading in these binding assays; the tips were hydrated for at least 30 min in PBS buffer, loaded with His-tagged integrin α2β1 (in PBS buffer for 180 s), then moved to baseline buffer to wash unbound His-tagged integrin α2β1 as baseline step (60 s), then associated with sequential diluted 2D-HFn or HFn (from 0.2 to 5 μM) with 200 uL binding buffer volume were placed in Black 96-well plates (association step, 720 s). Finally, the tips were moved to the baseline buffer for dissociation analysis (300 s); the association and dissociation profiles can be obtained for data fitting analysis with Octet DataAnalysis software (9.0.0.6.). The control study with tips in the absence of ferritins were also conducted in parallel, the results showed low nonspecific binding and the average values were subtracted for later kinetic binding analysis. The mathematical model used assumes a simple 1:1 stoichiometry, fitting only one analyte in solution binding to one binding site on the surface. A Savitzky-Golay filter was applied to smooth the data.

### Cellular binding of HFn and 2D-HFn

For FITC labeling, 50 μL of 2.5 mM FITC was added to 400 μL of 3.6 μM 2D-HFn (0.1 M in sodium bicarbonate buffer, pH 9) and incubated at 4 °C overnight in the dark. Unlabeled dye was removed by using a PD-10 column (GE Healthcare, Aurora, OH, USA). For flow cytometry analysis, U-87MG cells were harvested and resuspended in 100 µL of FITC-labeled 2D-HFn (40 µg mL^−1^) or FITC-labeled HFn (with the same amount of FITC as FITC-labeled 2D-HFn) for 30 min at 22 °C. After washing with PBS, the cells were analyzed with a BD FACS Aria III Sorter and ModFit LT version 3.25 software (BD Bioscience, San Jose, CA, USA). For confocal imaging, cells were seeded on a 4-well chamber slide (Thermo, Waltham, MA, USA). The next day, FITC-labeled 2D-HFn (20 µg mL^−1^) or FITC-labeled HFn (with the same amount of FITC as FITC-labeled 2D-HFn) was added to the medium followed by incubation for 30 min at 37 °C. After washing with PBS and fixation with 4% paraformaldehyde, the cell nuclei were stained with Hoechst 33342 (1 µg mL^−1^). Imaging was acquired with a Leica TCS SP5 confocal spectral microscope. For DOX cellular uptake experiments, cells were treated with DOX (5 µg mL^−1^) or DOX-loaded 2D-HFn-FITC (with the same amount of DOX) for various times. After washing with PBS and fixation with 4% paraformaldehyde, the cell nuclei were stained with Hoechst 33342, and images were acquired with a Leica TCS SP5 confocal spectral microscope. The filter sets for emission wavelengths of the FITC signal and DOX were 530 nm and 590 nm, respectively.

### Cellular uptake of 2D-HFn-DOX

Cells were seeded into a 96-well plate at the densities of 2 × 10^4^ cells per well. After overnight incubation, free DOX, HFn-DOX and 2D-HFn-DOX were added to the medium with a final concentration of DOX in each well of 2 μg mL^−1^, followed by incubation for selected time points. After washing with PBS and lysing with RIPA buffer, the DOX concentrations in the cell lysates were measured by their absorbance at 590 nm. The DOX standard curve was constructed with various concentrations of DOX in the untreated cell lysates. The cellular uptake of DOX at different time points is presented as the measured DOX amount in the cell lysates divided by the total protein amount in the cell lysate.

### In vitro transcytosis assay

Mouse BBB endothelial cells (ECs), bEnd.3, were seeded in the top Boyden chamber (Millicell® Hanging Cell Culture Insert) of a transwell (0.4-μm pore size) apparatus, and cells served as the receiving cells in the lower chamber in a 6-well plate to generate an in vitro BBB model. Approximately 1 × 10^6^ BBB ECs were seeded on a transwell plate and allowed to grow for 24 h, and the transendothelial electrical resistance (TEER) was recorded using an Epithelial Volt/Ohm Meter (EVOM^2^) (World Precision Instruments). 2.5 × 10^5^ U-87MG cells were seeded in a well of a 6-well plate and allowed to grow for 24 h. Subsequently, mouse BBB cells were cocultured with U-87MG cells for another 24 h. The transcytosis assay was performed by adding FITC-labeled HFn or 2D-HFn (approximately 100 μg of protein with an amount of FITC equivalent to 2 µg) to fresh culture media in the top (apical) chamber. FITC (2 μg) alone or cells alone were used as the controls. After 4 h of incubation, transcytosis of the HFn or 2D-HFn proteins was determined by collecting U-87MG cells from the bottom (basal) chamber. The amount of HFn/2D-HFn on the U-87MG cells in the basal chamber was analyzed by flow cytometry (BD FACSAria III) with FITC fluorescence using excitation at 490 nm and emission at 525 nm. The experiments were performed in triplicate.

### Cell cytotoxicity assay (MTT assay)

U-87MG cells were seeded in a 96-well plate at a density of 1 × 10^4^ cells per well. The next day, the cells were treated with various concentrations of 2D-HFn-DOX, HFn-DOX or free DOX. After 24 h, each well was washed with PBS and replaced with fresh medium. Then, MTT (5 mg mL^−1^) was added to the wells followed by incubation for 4 h. The purple formazan crystals were dissolved in acidified isopropanol (0.04 N HCl in isopropanol) and the absorbance was measured at 570 nm.

### Xenograft tumor model

Athymic male nude mice were obtained from BioLASCO Taiwan Co., Ltd. All animal experiments were performed according to a protocol approved by the Institutional Animal Care and Use Committee (IACUC) of National Taiwan University and Chang Gung Memorial Hospital, Linkou, Taiwan. U-87MG cells (5 × 10^6^) were prepared in 100 μL of PBS and subcutaneously injected into the front flanks or shoulder regions of 6–8-week-old male nude mice. The resultant tumors were allowed to grow for 3–5 weeks until they reached volumes of 200–500 mm.^3^ Tumor growth was monitored by caliper measurements of the perpendicular dimensions of each mass. The orthotopic U-87MG tumor implantation procedure was reported previously [47]. In brief, animals were anesthetized with 2% isoflurane gas and immobilized on a stereotactic frame. A sagittal incision was made through the skin overlying the calvarium, and a 27G needle was used to create a hole in the exposed cranium 1.5 mm anterior and 2 mm lateral to the bregma. Five microliters of the U-87MG cell suspension (1 × 10^5^ cells µL^−1^) was injected at a depth of 2 mm from the brain surface over a 1-min period, and the needle was withdrawn over 2 min. Tumor growth and the therapeutic effects of treatment were monitored longitudinally by magnetic resonance imaging (MRI) postimplantation.

### In vivo and ex vivo imaging

IRDye800 labeling of HFn protein was performed according to the manufacturer’s instructions. Briefly, 10.6 μL of 3.4 mM IRDye® 800CW NHS ester (LI-COR Biosciences, Lincoln City, NE, USA) was added to 200 μL of 9 µM 2D-HFn (0.1 M in phosphate buffer, pH 8.5). After incubation at 22 °C for 2 h in the dark, the unlabeled dye was removed by using a PD-10 column (GE Healthcare, Aurora, OH, USA). The amount of IRDye800 labeled on 2D-HFn was determined by measuring the absorbance at 780 nm using a SpectraMAX M3 Multi-Mode microplate reader (Molecular Devices, San Jose, CA, USA). For in vivo imaging, IRDye800-labeled 2D-HFn (6.4 mg kg^−1^ mice) was injected into the tail veins, and images were acquired with an IVIS Lumina II system (PerkinElmer, Waltham, MA, USA) at various time points, including 30 min, 1 h, 2 h and 24 h. The filter set with excitation and emission wavelengths of 675 nm and 760 nm, respectively, was used to measure the fluorescence signal of IRDye800. For ex vivo studies, 24 h after injection, the mice were sacrificed, and various organs were dissected. The IRDye800 signals of the tumors and other organs (brain, heart, lung, muscle, liver, pancreas, bone, kidney and spleen) were acquired and quantified by the IVIS Lumina II system.

### Therapeutic treatments

Subcutaneous brain tumor model: U-87MG tumor-bearing mice were randomly divided into four groups: the control group (saline), free DOX group (2 mg kg^−1^), 2D-HFn-DOX group (2 mg kg^−1^ DOX and 15 mg kg^−1^ 2D-HFn) and 2D-HFn group (15 mg kg^−1^). When tumor sizes were approximately 80 mm^3^, each group of mice was i.v. injected into the tail vein with the corresponding treatment dosage, which was performed every three days for a total of three injections. The tumor sizes and body weights were measured every two days for three weeks. Tumor sizes were calculated according to the formula: tumor volume = length × (width^2^)/2. After the mice were sacrificed, the tumors and several organs (heart, liver and kidney) of the mice were dissected, embedded in paraffin and stained with hematoxylin and eosin (H&E).

Transcranial brain tumor model: For the orthotopic tumor model, three experimental arms were arranged to validate the therapeutic effects of 2D-HFn-DOX. There was a control group (injected with saline), free DOX group (1 mg kg^−1^), HFn-DOX (1 mg kg^−1^) and 2D-HFn-DOX group (1 mg kg^−1^ DOX). When the animals developed viable and suitably sized tumor lesions as characterized by MR imaging, this was regarded as the starting point that was used to evaluate the effects of the treatments. The in vivo treatment responses from each subgroup were simultaneously monitored with standard ^18^F-fluorodeoxyglucose (FDG)-positron emission tomography (PET) scans and MR images. The survival time of each treated group were drawn for therapeutic effect comparison studies.

### Statistical analysis

All statistical analyses were performed using unpaired Student’s *t*-tests or two-way ANOVA followed by Tukey’s HSD test.

## Supplementary Information

**Additional file 1.** Additional figures and tables.
