# Supplementary material for: Integrin α2β1-targeting ferritin nanocarrier traverses the blood–brain barrier for effective glioma chemotherapy
Source: J Nanobiotechnology. 2021 Jun 13;19:180. doi: 10.1186/s12951-021-00925-1 (PMC8201891; doi:10.1186/s12951-021-00925-1)
Supplement: Supplementary file 1 — Additional file 1. Additional figures and tables. [file 12951_2021_925_MOESM1_ESM.pdf]

Additional file 1

Additional figures and tables

Integrin $\alpha_2\beta_1$-Targeting Ferritin Nanocarrier Traverses the Blood-Brain Barrier for Effective Glioma Chemotherapy

Chiun-Wei Huang[1], Chia-Pao Chuang[2#], Yan-Jun Chen[2#], Hsu-Yuan Wang[2#], Jia-Jia Lin[1], Chiung-Yin Huang[3], Kuo-Chen Wei[345] and Feng-Ting Huang[2]†

[1]Center for Advanced Molecular Imaging and Translation (CAMIT), Department of Medical Research, Chang Gung Memorial Hospital, Linkou, Taiwan.

[2]Department of Biochemical Science and Technology, College of Life Science, National Taiwan University, Taipei, Taiwan.

[3]Department of Neurosurgery, Chang Gung Memorial Hospital, Linkou, Taiwan

[4]Department of Neurosurgery, New Taipei Municipal TuCheng Hospital, New Taipei City, Taiwan

[5]School of Medicine, Chang Gung University, Taoyuan, Taiwan

*These authors contributed equally to this work.

†**Corresponding author**: Feng-Ting Huang, Ph.D. Department of Biochemical Science and Technology, College of Life Science, National Taiwan University. AC2-414, No.1, Sec. 4, Roosevelt Rd., Taipei, Taiwan 106. Tel. No.: 886-2-3366-4083, Fax. No.: 886-2-3366-2271. email: fthuang@ntu.edu.tw.

Additional Figures

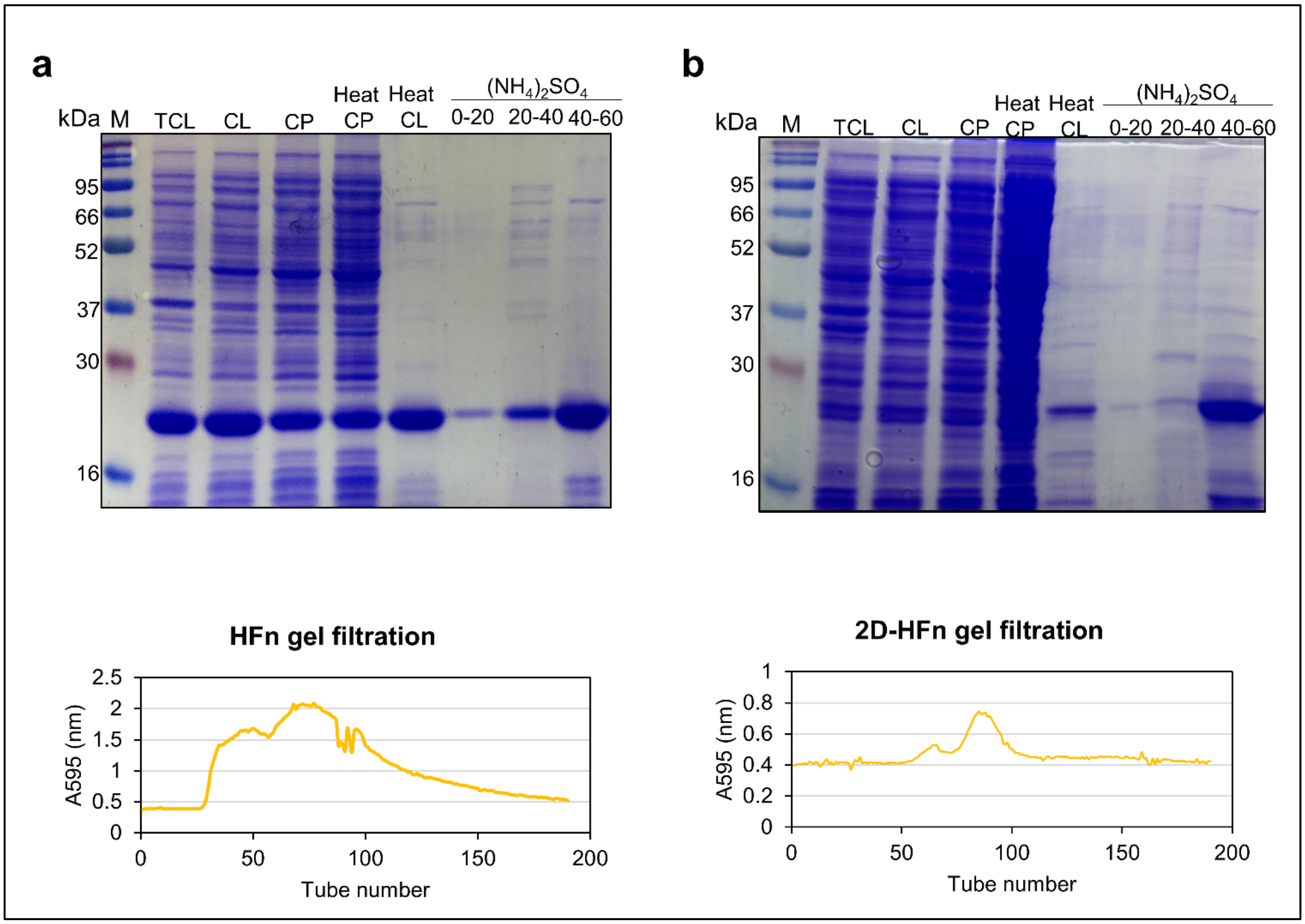

Figure S1. Purification of HFn and 2D-HFn. The CBR staining of the SDS-PAGE of each purification step of protein and the gel filtration chromatography of each protein (Sephacryl S-300 HR column). **a** HFn, and **b** 2D-HFn.

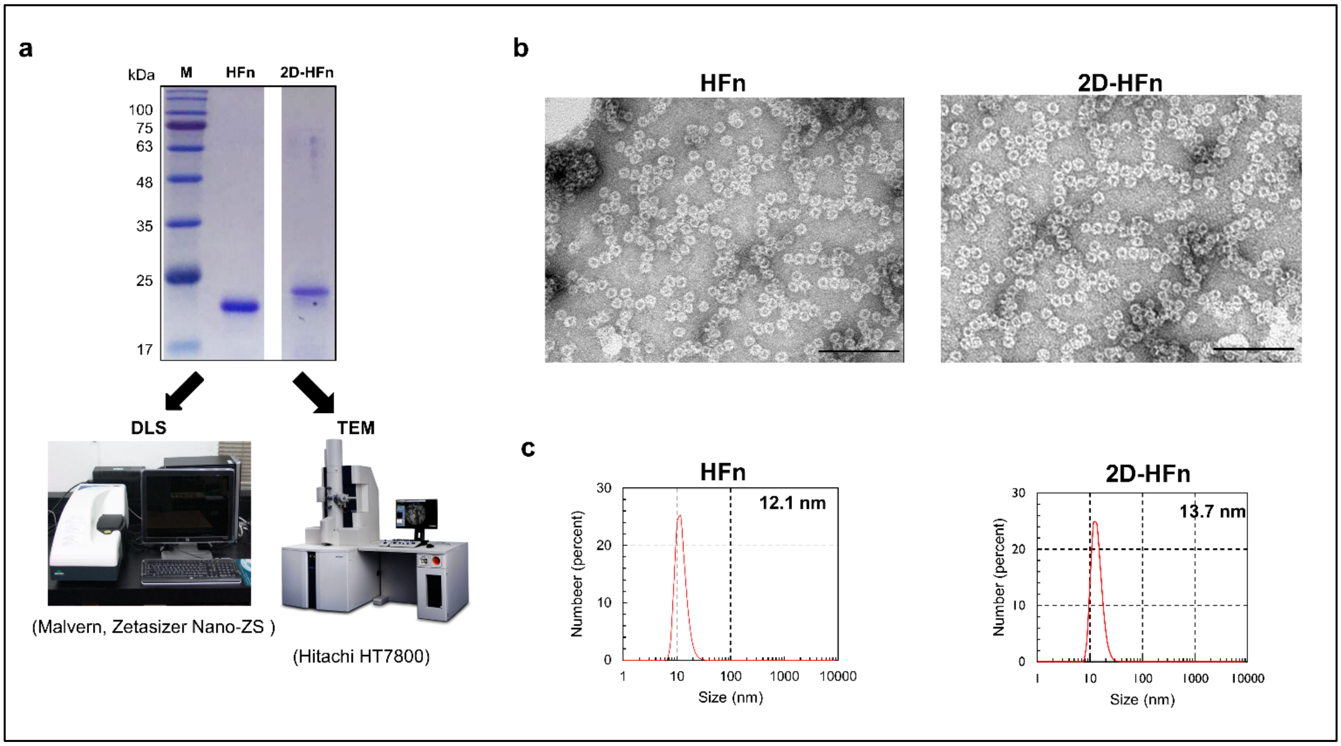

Figure S2. The recombinant ferritin proteins formed the hallow globular structure. a The purified HFn and 2D-HFn were resolved on 12% SDS-PAGE followed by CBR staining. **b** Images of the hollow cage structures of the HFn and 2D-HFn nanoparticles were acquired by TEM. Scale bar, 100 nm. **c** The average diameters of the HFn and 2D-HFn nanoparticles were 12.1 and 13.7 nm, respectively, as measured by DLS analysis.

a

Type	Yield (%)	Loading efficiency (%)	Number of drug/ nanoparticle
HFn	18.3	11.0	109
2D-HFn	24.5	61.7	458

b

- For DOX encapsulation, 1 mg HFn/2D-HFn and 200 μg DOX were utilized initially. After encapsulation, the recovered protein amount and DOX in the protein were measured for calculation of the yield and loading efficiency.
- The loading efficiency = [weight of DOX loaded in protein/total weight of added DOX] × 100%.

  Yield = [weight of final protein /total weight of added protein] × 100%.

Type	Yield (%)	Loading efficiency (%)
HFn	18.3	11.0
2D-HFn	24.5	61.7

	Molecule weight (MW)
DOX	580 Da
HFn	504 kDa (21 kDa x 24 subunits)
2D-HFn	528 kDa (22 kDa x 24 subunits)

- The DOX number on each nanoparticle = $\frac{\text{[DOX molecule number loaded in protein]}}{\text{[HFn or 2D-HFn molecule number]}}$

$$= \frac{\text{[DOX amount loaded in protein / DOX MW]}}{\text{[the final protein amount / MW of HFn or 2D-HFn]}} = \frac{[200\ \mu\text{g x Loading efficiency / DOX MW}]}{\text{[1 mg x Yield / MW of HFn or 2D-HFn]}}$$

- Hence

The DOX number on HFn nanoparticle = $\frac{[200\ \mu\text{g x 11\% / 580 Da}]}{\text{[1 mg x 18.3\% / 504 kDa]}} = 109$

The DOX number on 2D-HFn nanoparticle = $\frac{[200\ \mu\text{g x 61.7\% / 580 Da}]}{\text{[1 mg x 24.5\% / 528 kDa]}} = 458$

Figure S3. Encapsulation efficiency of DOX into 2D-HFn and HFn by the pH-mediated disassembly and reassembly method. a. Yield, loading efficiency and the DOX number on the HFn/2D-HFn nanoparticles. **b**. Calculation for the DOX number on the HFn/2D-HFn nanoparticles.

Ex Vivo fluorescent imaging and histological analysis

To investigate the expression level of integrin receptor and neovasculature histologically, the immunohistochemistry and fluorescent microscopic imaging were used to confirm the colocalization of activated integrins and accumulation of the fluorescent dye tagged 2D-HFn particles. Mouse tumors were excised after IV-injection of IRDye800-2D-HFn, and the collected dissected U-87MG brain tumor tissues were embedded in OCT compound (Tissue-Tek), snap-frozen in a liquid nitrogen filled chamber, and then cryo-sectioned with a thickness of 10 μm. The fluorescent images were taken by a Leica MZ75 (Leica, Inc., Germany) high-performance stereomicroscope equipped with 2.5 × plano objective. Staining for integrins and CD31 was done by immunohistochemistry on histologic sections that were fixed in methanol and pre-incubated with 0.3% H_2O_2 to block endogenous peroxidase activity. The sections were then blocking by 1% BSA for 1 h and were incubated with anti-integrin α2β1 monoclonal antibodies (ab30483, dilution 1:50 in Tris buffer) and CD31/PECAM1 Rabbit monoclonal antibodies (A19014, dilution 1:50 in Tris buffer) at 4 °C overnight. After washing with TBS, the slides were incubated with HRP-conjugated goat anti-mouse IgG and anti-rabbit secondary antibody (dilution, 1:200 in Tris buffer) for 1 h at room temperature. The reaction was developed in 3, 3'-diamino benzidine (Sigma).

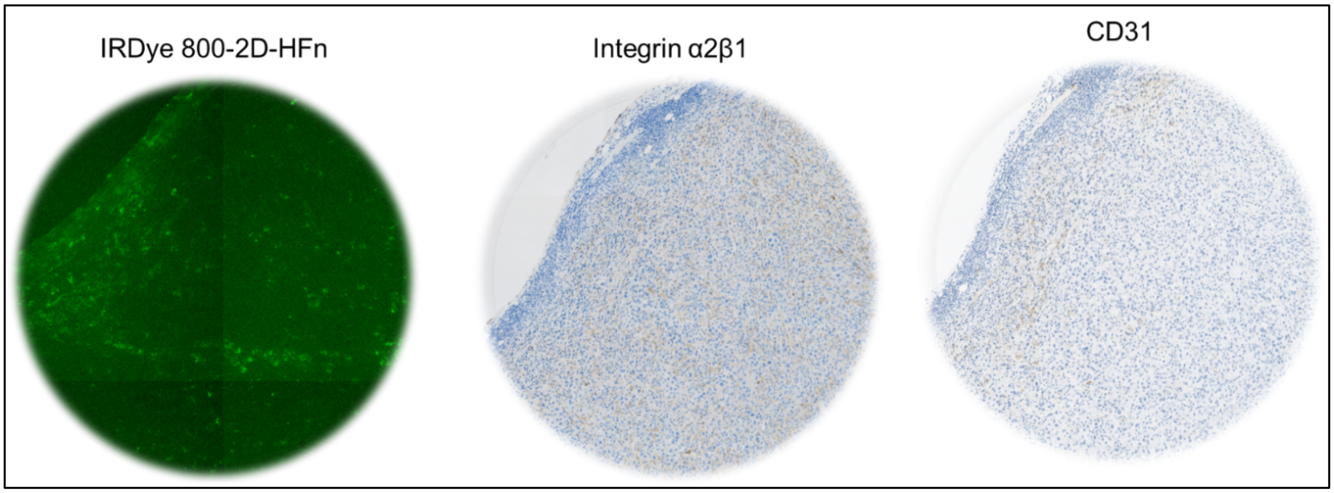

Figure S4. Ex Vivo fluorescent imaging and histological analysis. The intensity of 2D-HFn nanoparticle in digitalized fluorescent imaging matches with the integrin-stained histological result. In addition, the high colocolization of integrin α2β1 and CD31 staining results (particularly in peritumoral regions), confirming the potential role of integrin α2β1during tumor angiogenesis process.

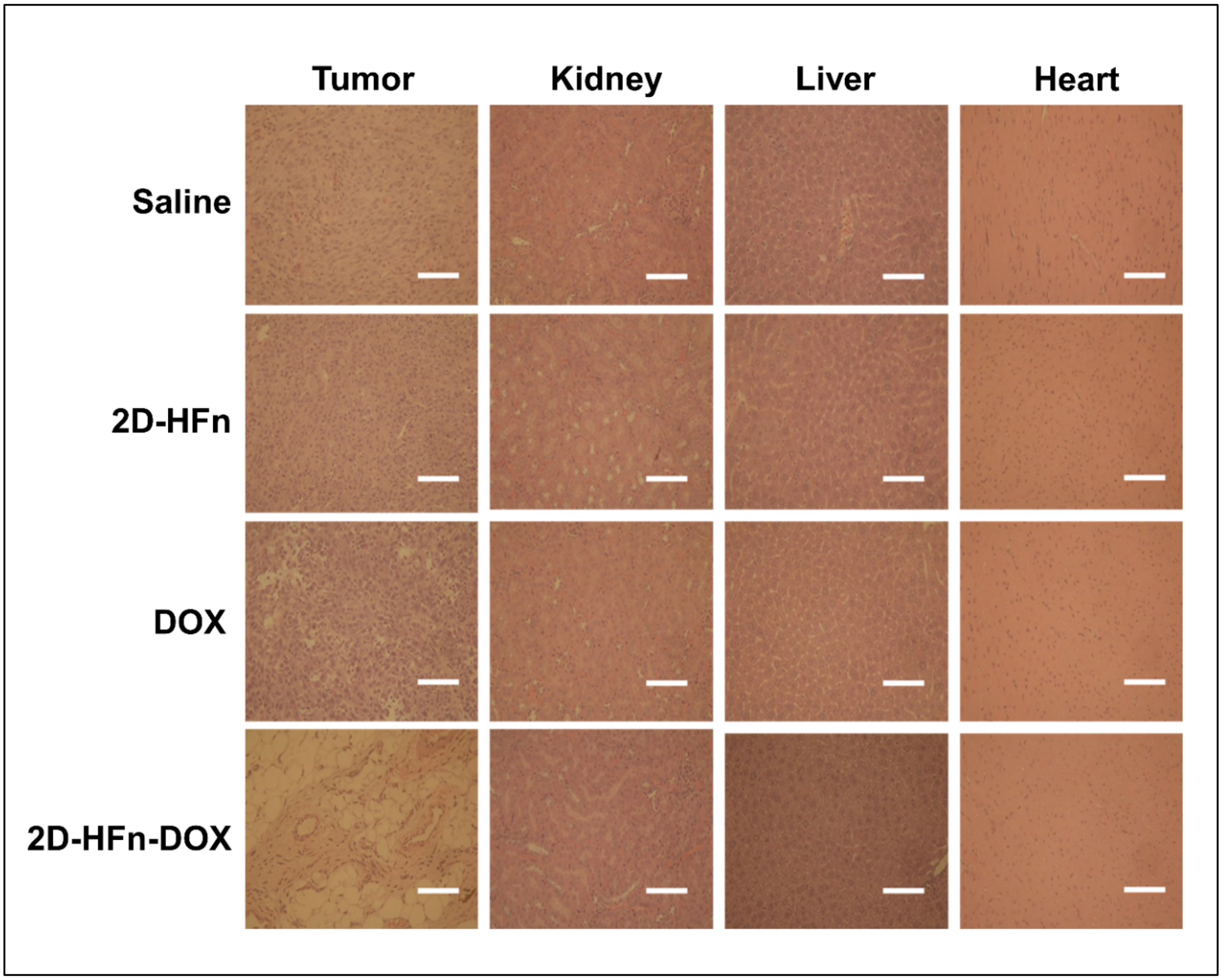

Figure S5. The H&E staining of sections of the tumor and various organs (heart, liver and kidneys) of different treatments in subcutaneous tumor mouse models. Scale bar, 0.2 mm.

Figure S6. Integrin α2 (ITGA2) and integrin β1 (ITGB1) expression were up-regulated in pancreatic cancer in clinical cancer samples in four datasets from the Oncomine online microarray database. The mRNA level of integrin α2 (ITGA2) and integrin β1 (ITGB1) in clinical pancreatic cancer tissues and normal pancreas tissues were acquired and analyzed from the Oncomine online microarray database. A box-and-whisker plot that represents ITGA2 and ITGB1 expression from primary tumors compared with normal specimens in four datasets from the Oncomine database. The horizontal top and bottom lines of each box represented the 75th and the 25th percentile, respectively. The band in the box is the median value. Horizontal lines above and below the box represented the 90th and the 10th percentile, respectively. The dots above the 90th percentile and below the 10th percentile represented the maximum and minimum values, respectively. From four pancreas datasets, integrin α2 (ITGA2) and integrin β1 (ITGB1) were up-regulated in tumor tissue specimens in comparison to normal tissues. **a** The Badea Pancreas compromises 78 specimens, including 39 normal pancreas specimens and 39 pancreatic ductal adenocarcinoma specimens. **b** The Pei Pancreas compromises 52 specimens, including 16 normal pancreas specimens and 36 pancreatic carcinoma specimens. **c** The Iacobuzio-Donahue Pancreas 2 compromises 15 specimens, including 4 normal pancreas specimens and 11 pancreatic adenocarcinoma specimens. **d** The Logsdon Pancreas compromises 15 specimens, including 5 normal pancreas specimens and 10 pancreatic adenocarcinoma specimens. *** $P < 0.001$.

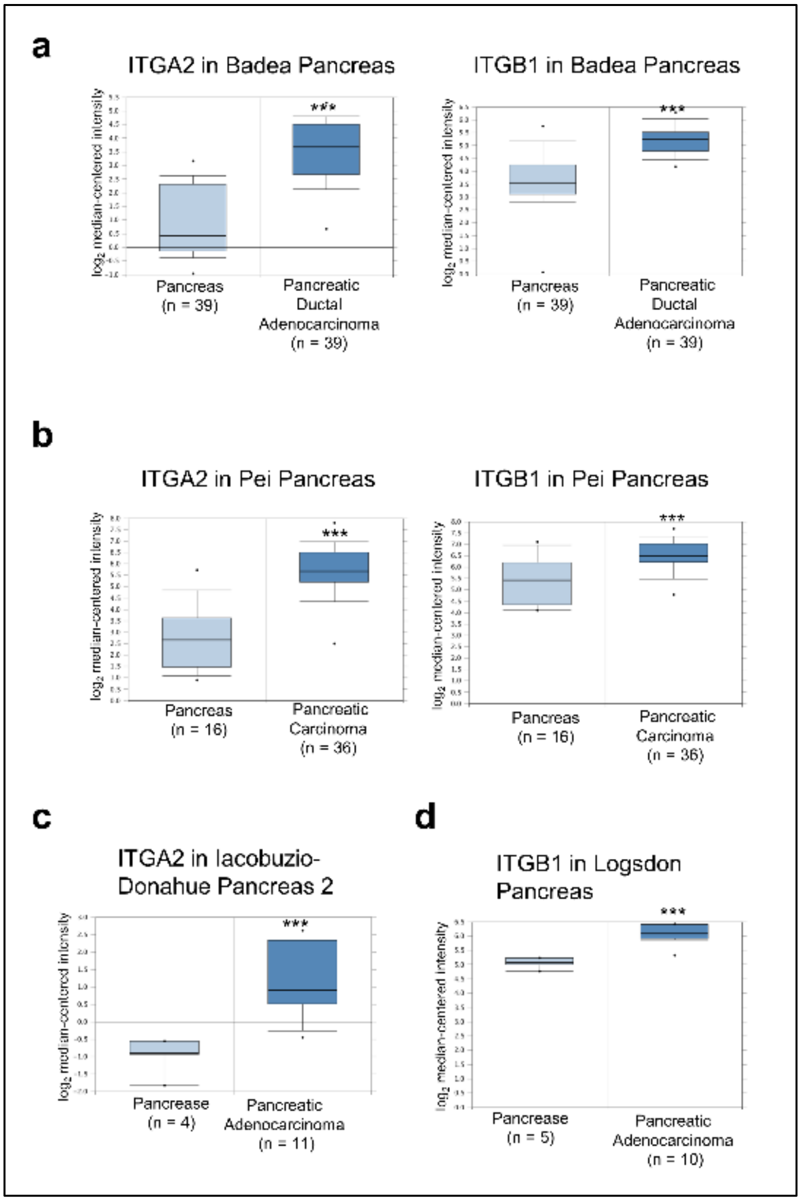

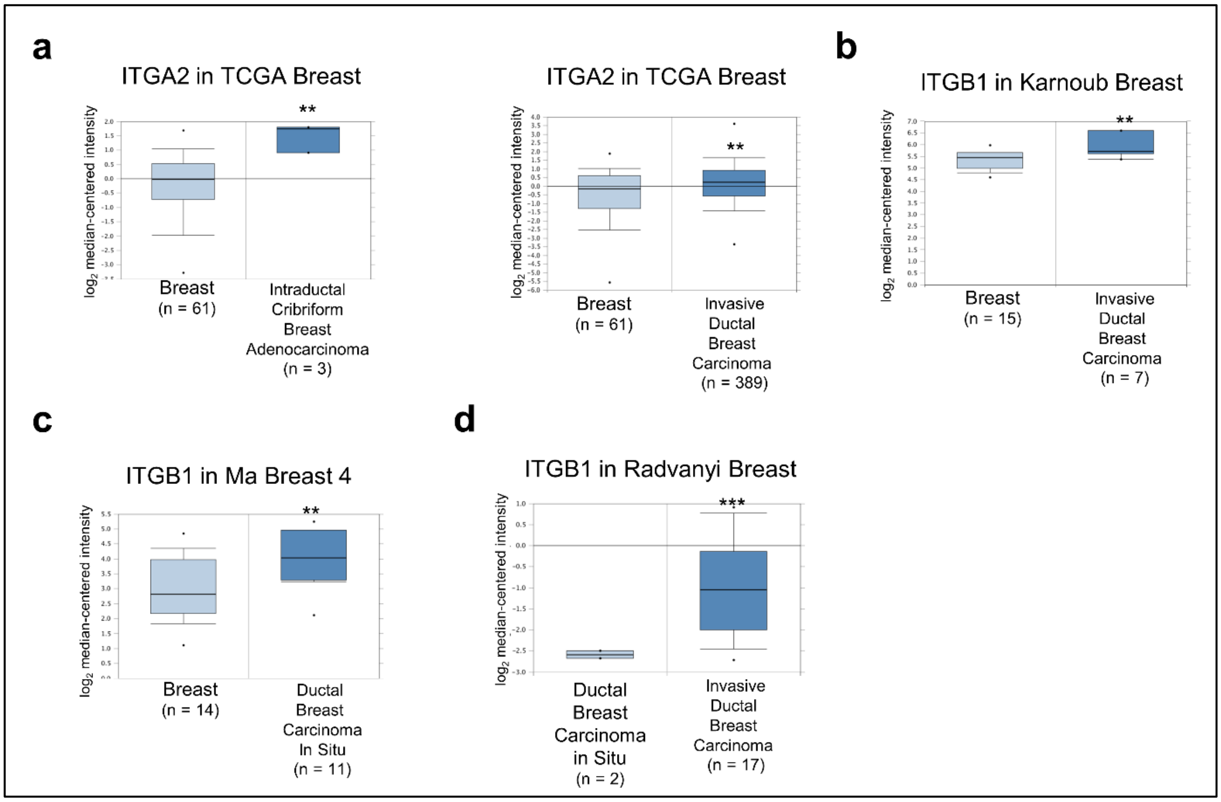

Figure S7. Integrin α2 (ITGA2) and Integrin β1 (ITGB1) expression were up-regulated in breast cancer in clinical cancer samples in four datasets from the Oncomine online microarray database. The mRNA level of integrin α2 (ITGA2) and integrin β1 (ITGB1) in clinical breast cancer tissues and normal breast tissues were acquired and analyzed from the Oncomine online microarray database. A box-and-whisker plot that represents ITGA2 and ITGB1 expression from primary tumors compared with normal specimens in several datasets from the Oncomine database. The horizontal top and bottom lines of each box represented the 75th and the 25th percentile, respectively. The band in the box is the median value. Horizontal lines above and below the box represented the 90th and the 10th percentile, respectively. The dots above the 90th percentile and below the 10th percentile represented the maximum and minimum values, respectively. From four breast cancer datasets, integrin α2 (ITGA2) and integrin β1 (ITGB1) were up-regulated in aggressive tumor tissue specimens in comparison to normal tissues and other subtypes of breast cancer. **a** The TCGA Breast compromises 453 specimens, including 61 normal breast specimens, 3 intraductal cribriform breast adenocarcinoma and 389 invasive ductal breast carcinoma specimens. **b** The Karnoub Breast compromises 22 specimens, including 15 normal breast specimens and 7 invasive ductal breast carcinoma specimens. **c** The Ma Breast 4 compromises 25 specimens, including 14 normal breast specimens and 11 ductal breast carcinoma in situ specimens. **d** The Radvanyi Breast compromises 19 specimens, including 2 ductal breast carcinoma in situ specimens and 17 invasive ductal breast carcinoma specimens. ** $P < 0.01$, *** $P < 0.001$.

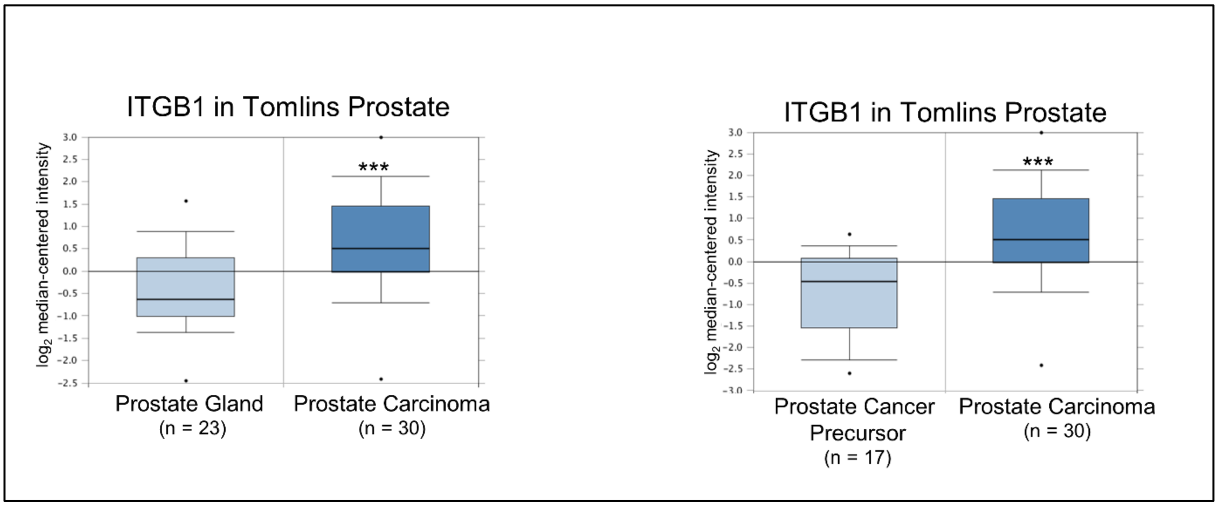

Figure S8. Integrin β1 (ITGB1) expression was up-regulated in prostate cancer in clinical cancer samples in Tomlins Prostate dataset from the Oncomine online microarray database. The mRNA level of integrin β1 (ITGB1) in clinical prostate cancer tissues and normal prostate tissues were acquired and analyzed from the Oncomine online microarray database. A box-and-whisker plot that represents ITGB1 expression from primary tumors compared with normal specimens in Tomlins Prostate dataset from the Oncomine database. The horizontal top and bottom lines of each box represented the 75th and the 25th percentile, respectively. The band in the box is the median value. Horizontal lines above and below the box represented the 90th and the 10th percentile, respectively. The dots above the 90th percentile and below the 10th percentile represented the maximum and minimum values, respectively. From the Tomlins Prostate datasets, integrin β1 (ITGB1) was up-regulated in tumor tissue specimens in comparison to normal tissues. The Tomlins Prostate compromises 70 specimens, including 23 normal prostate specimens, 17 specimens from prostate cancer precursor and 30 prostate cancer specimens. *** $P < 0.001$.

Additional Tables

Table S1. Integrin α2 (ITGA2) and integrin β1 (ITGB1) expression were up-regulated in clinical GBM samples from three datasets

	Dataset	Tumor specimen type (number)	Compared specimen (number)	Gene	Fold change (+)	*p*-value	GEO number	Gene number
1	Bredel Brain 2	Glioblastoma (31)	Normal Brain (4)	ITGA2	2.227	1.19E-6	GSE2223	14836
				ITGB1	2.43	4.43E-4		
2	TCGA Brain	Glioblastoma (542)	Normal Brain (10)	ITGA2	1.932	1.66E-5	-	12624
				ITGB1	4.437	5.00E-12		
3	Sun Brain	Glioblastoma (81)	Normal Brain (23)	ITGA2	1.432	1.39E-4	GSE4290	19574
				ITGB1	2.461	3.87E-11		
4	Bredel Brain 2	Glioblastoma (31)	Astrocytoma (3)<br>Mixed Glioma (6)<br>Oligodendroglial Tumor (8)	ITGA2	3.259	4.23E-9	GSE2223	14836
				ITGB1	1.968	9.11E-5	GSE2223	14836
5	Freije Brain	Glioblastoma (59)	Astrocytoma (8)<br>Mixed Glioma (7)<br>Oligodendroglial Tumor (11)	ITGA2	1.58	3.41E-6	GSE4412	17779
				ITGB1	1.569	1.57E-7	GSE4412	17779
6	Sun Brain	Glioblastoma (81)	Astrocytoma (26)<br>Oligodendroglial Tumor (50)	ITGA2	1.490	8.15E-10	GSE4290	19574
				ITGB1	1.662	4.72E-11	GSE4290	19574
7	Sun Brain	Diffuse Astrocytoma (7)	Normal Brain (23)	ITGB1	2.565	1.29E-4	GSE4290	19574

Table S2. Integrin α2 (ITGA2) and integrin β1 (ITGB1) expression were up-regulated in clinical pancreatic cancer samples from four datasets

	Dataset	Tumor specimen type (number)	Compared specimen (number)	Gene	Fold change (+)	*p*-value	GEO number	Gene number
1	Badea Pancreas	Pancreatic Ductal Adenocarcinoma (39)	Normal Pancreas (39)	ITGA2	6.394	2.22E-16	GSE15471	19574
				ITGB1	2.954	1.77E-12		
2	Pei Pancreas	Pancreatic Carcinoma (36)	Normal Pancreas (16)	ITGA2	8.163	3.41E-8	GSE16515	19574
				ITGB1	2.227	1.86E-4		
3	Iacobuzio-Donahue Pancreas 2	Pancreatic Adenocarcinoma (11)	Normal Pancreas (4)	ITGA2	4.304	1.84E-4	GSE3654	14380
5	Logsdon Pancreas	Pancreatic Adenocarcinoma (10)	Normal Pancreas (5)	ITGB1	1.988	2.88E-6	-	5338

Table S3. Integrin α2 (ITGA2) and integrin β1 (ITGB1) expression were up-regulated in clinical breast cancer samples from five datasets

	Dataset	Tumor specimen type (number)	Compared specimen (number)	Gene	Fold change (+)	*p*-value	GEO number	Gene number
1	TCGA Breast	Intraductal Cribriform Breast Adenocarcinoma (3)	Normal Breast (61)	ITGA2	3.348	0.006	-	20423
2	TCGA Breast	Invasive Ductal Breast Carcinoma (389)	Normal Breast (61)	ITGA2	1.596	0.001	-	20423
3	Karnoub Breast	Invasive Ductal Breast Carcinoma (7)	Normal Breast (15)	ITGB1	1.570	0.006	GSE8977	19574
4	Ma Breast 4	Ductal Breast Carcinoma In situ (11)	Normal Breast (14)	ITGB1	2.039	0.007	GSE14548	19139
5	Radvanyi Breast	Invasive Ductal Breast Carcinoma (17)	Ductal Breast Carcinoma In situ (2)	ITGB1	3.277	8.27E-6	GSE1477	16,775
